# Genome-wide investigation of persistence with methotrexate treatment in early rheumatoid arthritis

**DOI:** 10.1093/rheumatology/kead301

**Published:** 2023-06-16

**Authors:** Anton Öberg Sysojev, Saedis Saevarsdottir, Lina-Marcela Diaz-Gallo, Gilad N Silberberg, Lars Alfredsson, Lars Klareskog, Eva Baecklund, Lena Björkman, Alf Kastbom, Solbritt Rantapää-Dahlqvist, Carl Turesson, Ingileif Jonsdottir, Kari Stefansson, Thomas Frisell, Leonid Padyukov, Johan Askling, Helga Westerlind

**Affiliations:** Clinical Epidemiology Division, Department of Medicine Solna, Karolinska Institute, Stockholm, Sweden; Clinical Epidemiology Division, Department of Medicine Solna, Karolinska Institute, Stockholm, Sweden; Faculty of Medicine, School of Health Sciences, University of Iceland, Reykjavik, Iceland; deCODE Genetics Inc, Reykjavik, Iceland; Division of Rheumatology, Department of Medicine Solna, Karolinska Institute and Karolinska University Hospital, Stockholm, Sweden; Center for Molecular Medicine, Department of Medicine Solna, Karolinska Institute, Stockholm, Sweden; Division of Rheumatology, Department of Medicine Solna, Karolinska Institute and Karolinska University Hospital, Stockholm, Sweden; Center for Molecular Medicine, Department of Medicine Solna, Karolinska Institute, Stockholm, Sweden; Institute of Environmental Medicine (IMM), Karolinska Institute, Stockholm, Sweden; Division of Rheumatology, Department of Medicine Solna, Karolinska Institute and Karolinska University Hospital, Stockholm, Sweden; Center for Molecular Medicine, Department of Medicine Solna, Karolinska Institute, Stockholm, Sweden; Department of Medical Sciences, Rheumatology, Uppsala University, Uppsala, Sweden; Department of Rheumatology and Inflammation Research, University of Göteborg, Göteborg, Sweden; Department of Biomedical and Clinical Sciences, Linköping University, Linköping, Sweden; Department of Public Health and Clinical Medicine, Rheumatology, Umeå University, Umeå, Sweden; Department of Clinical Sciences, Malmö, Lund University, Malmö, Sweden; Faculty of Medicine, School of Health Sciences, University of Iceland, Reykjavik, Iceland; deCODE Genetics Inc, Reykjavik, Iceland; Faculty of Medicine, School of Health Sciences, University of Iceland, Reykjavik, Iceland; deCODE Genetics Inc, Reykjavik, Iceland; Clinical Epidemiology Division, Department of Medicine Solna, Karolinska Institute, Stockholm, Sweden; Division of Rheumatology, Department of Medicine Solna, Karolinska Institute and Karolinska University Hospital, Stockholm, Sweden; Center for Molecular Medicine, Department of Medicine Solna, Karolinska Institute, Stockholm, Sweden; Clinical Epidemiology Division, Department of Medicine Solna, Karolinska Institute, Stockholm, Sweden; Department of Medical Sciences, Rheumatology, Uppsala University, Uppsala, Sweden; Clinical Epidemiology Division, Department of Medicine Solna, Karolinska Institute, Stockholm, Sweden

**Keywords:** RA, MTX, persistence, heritability, genetic polymorphism, predictors, biomarkers

## Abstract

**Objectives:**

To investigate the influence of genetic factors on persistence with treatment of early RA with MTX monotherapy.

**Methods:**

We conducted a genome-wide association study (GWAS) in a sample of 3902 Swedish early-RA patients initiating MTX in DMARD monotherapy as their first-ever DMARD. The outcome, short- and long-term MTX treatment persistence, was defined as remaining on MTX at 1 and at 3 years, respectively, with no additional DMARDs added. As genetic predictors, we investigated individual SNPs, and then calculated a polygenic risk score (PRS) based on SNPs associated with RA risk. The SNP-based heritability of persistence was estimated overall and by RA serostatus.

**Results:**

No individual SNP reached genome-wide significance (*P* < 5 × 10^−8^), either for persistence at 1 year or at 3 years. The RA PRS was not significantly associated with MTX treatment persistence at 1 year [relative risk (RR) = 0.98 (0.96–1.01)] or at 3 years [RR = 0.96 (0.93–1.00)]. The heritability of MTX treatment persistence was estimated to be 0.45 (0.15–0.75) at 1 year and 0.14 (0–0.40) at 3 years. The results in seropositive RA were comparable with those in the analysis of RA overall, while heritability estimates and PRS RRs were attenuated towards the null in seronegative RA.

**Conclusion:**

Despite being the largest GWAS on an MTX treatment outcome to date, no genome-wide significant associations were detected. The modest heritability observed, coupled with the broad spread of suggestively associated loci, indicate that genetic influence is of polygenic nature. Nevertheless, MTX monotherapy persistence was lower in patients with a greater genetic disposition, per the PRS, towards RA.

Rheumatology key messages

- Methotrexate treatment persistence in early RA has a modest genetic component of polygenic nature.
- High genetic predisposition towards RA is inversely associated with methotrexate treatment persistence.

## Introduction

RA is a chronic, inflammatory disorder primarily manifesting as inflammatory arthritis [1, 2]. To prevent irreversible joint damage and to improve long-term prognosis, early and efficient treatment is key [3, 4]. Despite the wealth of available DMARDs, most treatment guidelines recommend MTX as first-line DMARD monotherapy in newly diagnosed patients [5–7]. However, actual treatment response is highly variable; a significant proportion of patients fail to reach treatment targets, thus requiring treatment modifications or discontinuation of MTX [8–10]. We have previously demonstrated that, at 1 year after RA diagnosis and treatment initiation with MTX in DMARD monotherapy, one out of three patients no longer remain on this treatment regimen [10, 11]. Therefore, efficient predictors of persistence with MTX as first-line treatment would be of great clinical importance.

Genetic variation is one of the key components through which the observed variability in treatment outcome might be explained [11]. Genetic variants such as single nucleotide polymorphisms (SNPs) are measurable prior to treatment start, and would provide ample opportunities for use as predictive biomarkers to aid a priori stratification with respect to risk of treatment failure. However, despite nearly two decades of research, no genetic predictors of MTX treatment outcomes have yet been well validated [12–14]. Many studies have been undertaken on a number of outcome measures investigating a broad number of SNPs, but the results have often been contradictory and difficult to replicate. Attempts to aggregate results through meta-analyses have provided suggestive evidence for a handful of SNPs associated with MTX treatment outcomes, although individual effects have generally been weak and consequently of poor clinical relevance, with findings rarely concordant between studies [15–17]. Explanations for these results have been proposed, including low statistical power due to small study samples, a narrow focus on SNPs involved in the metabolic pathways of MTX, and low heritability of the studied MTX outcomes.

Mitigating some of the limitations of previous studies, we performed a genome-wide association study (GWAS) on treatment persistence with MTX in DMARD monotherapy among Swedish patients newly diagnosed with RA. Here, the outcome of treatment persistence acts as a clinically relevant proxy both for having sufficiently controlled disease activity and for sufficient tolerance of the drug. We further assessed whether established RA-risk SNPs additionally predict treatment persistence with MTX by testing for an association between a polygenic risk score (PRS) for RA risk and MTX treatment persistence. Additionally, to quantify the influence of genetics on the variation in MTX treatment persistence, we also estimated the SNP-based heritability of the outcome under study.

## Methods

### Setting and data sources

Our primary analysis cohort consisted of early-RA patients receiving MTX in DMARD monotherapy as their first RA treatment, identified in the Epidemiological Investigation of RA (EIRA) biobank or the Swedish Rheumatology Quality Register’s (SRQ) biobank (SRQb). EIRA, a case–control study established in 1996, currently contains >5000 incident RA cases from the middle and southern regions of Sweden [18]. SRQ is a nationwide quality register established in the mid-1990s used by most rheumatologists in Sweden to monitor the disease course of patients as part of standard care. SRQb is an ongoing research project currently holding blood samples from >5500 RA patients, with either early RA or starting a bDMARD.

### Study population

Study inclusion was restricted to early-RA patients starting MTX between 1996 and 2020 included into any of the studies prior to 2018, who consented to donation of blood for genotyping. Early-RA patients were defined as those starting MTX no more than 12 months after their RA diagnosis, with the RA diagnosis made by the treating rheumatologist, either based on the ACR 1987 or EULAR 2010 criteria [19, 20]. Patients were only included if they were treated with MTX as their first DMARD, in DMARD monotherapy. The latter was defined as not being prescribed any non-MTX DMARD within 30 days of the first MTX prescription, but allowed MTX in combination with oral/i.a. glucocorticoids and/or NSAIDs. This study was approved by the Stockholm ethical review board (DNR 96–174, DNR 2006/476–31/4 and DNR 2012/2070–31/2).

### Outcome

The primary outcome was MTX treatment persistence, defined as a binary variable in two versions: persistence at 1 and 3 years (i.e. at 365 days and 1096 days after treatment start, respectively), as in our previous studies [10, 11]. In short, a patient was considered to be MTX treatment persistent if MTX was continuing at the time-point of evaluation, with no other non-MTX DMARD prescriptions being made during the period following MTX initiation and up until the evaluation time-point. Further details on the treatment outcome have been previously described [10, 11].

### Genetic data

Samples from study participants were genotyped at deCODE genetics Inc., Iceland, on the Illumina Infinium Global Screening Array (GSA). Genotyped data for both cohorts were combined and imputed against the 1000 Genomes Phase III reference panel [21]. Prior to statistical analysis, data were processed through a stringent quality control (QC). Further details on genotyping, imputation and QC can be found in the Supplementary Note, available at *Rheumatology* online.

### Statistical analysis

The GWASs were performed using an additive logistic regression model over each autosomal SNP, for MTX treatment persistence at 1 and 3 years, respectively. Covariates adjusted for included sex, age, and the first *k* principal components estimated from the SNP data. Here, *k* was chosen from visual inspection of the principal component elbow plots. *P*-values of <5 × 10^–8^ were considered to have genome-wide significance; *P*-values of <5 × 10^–5^ were considered to have suggested associations. Association analysis was performed using PLINK [22, 23].

We used a PRS to test for an association between genetic risk factors for RA and MTX treatment persistence status. As training data, we used publicly available GWAS data on risk of RA [24], including all available SNPs passing a method-specific QC ascertaining compliance with methodological assumptions (Supplementary Note, available at *Rheumatology* online) [25]. The European subcohort of the 1000 Genomes Phase III data [21] was used as a linkage disequilibrium (LD) reference panel, after filtering out individuals and SNPs per the same QC pipeline as for the GWASs (Supplementary Note, available at *Rheumatology* online). Furthermore, to alleviate computational burden, a subset of SNPs exhibiting extreme LD (i.e. *r*^2^ ≥ 0.99) were pruned from the reference panel data. Construction of the PRS was performed in R (v4.1.2) [26], using LDpred2-auto [25] as implemented in the ‘bigsnpr’ package (v1.8.1). In short, LDpred2 uses SNP effect estimates from GWAS data with LD reference panel data to estimate posterior mean causal effect sizes that are subsequently used as weights within the PRS. As a result, no selection of SNPs or filtering past a *P*-value threshold is necessary when estimating the PRS weights, and all SNPs passing QC can be included, generally leading to improved performance over standard pruning-and-thresholding approaches [25, 27]. Validity of the PRS was ascertained by testing it for an association with the outcome of RA, using logistic regression [odds ratio (OR) = 1.87, 95% CI 1.82–1.92], in a population consisting of our study cohort and ∼3000 RA-free controls available from EIRA.

Association testing of the PRS for MTX treatment persistence was done by log-binomial regression, both crude and adjusted for sex, age, and genetic ancestry per the first *k* principal components, the number *k* taken to match that used within the GWAS of the corresponding outcome. To further elucidate the relationship between the PRS and the persistence outcomes, an additional analysis was performed after categorizing the continuous PRS based on the standard normal quintiles, comparing patients within each category against those within the lowest quintile using log-binomial regression. Regression analyses were performed in R (v4.1.2) [26].

Estimation of the SNP-based heritability was performed with genomic restricted maximum likelihood (GREML), as implemented in the GCTA software (v1.93.2b) [28, 29]. To account for using a binary outcome, heritability estimates were transformed and presented on the liability scale [28, 30], using 0.66 and 0.50 as prevalence of persistence at 1 year and 3 years, respectively, based on frequencies observed in previous studies within the same study population [10, 11]. Both crude and adjusted (age, sex and ancestry) estimates were calculated. Out-of-bounds CIs were truncated to 0/1 and are reported with an asterisk.

### Seropositive and seronegative subcohort analysis

In addition to the analyses described above, we performed similar analyses in seropositive and seronegative subcohorts, constructed by stratifying our primary analysis cohort by RA serostatus. Seropositivity was defined as positivity for either ACPA or RF. Patients negative for both autoantibodies were classified as seronegative. In EIRA, ACPA status was determined by screening of baseline sera for IgG anti-CCP2 using CCPlus^®^ ELISA (Euro-Diagnostica AB, Malmö, Sweden) according to the manufacturer’s instructions. Positivity was defined as ≥25 arbitrary-units/ml [31]. In SRQb, participants were screened for anti-CCP2 using the EliA^TM^ CCP assay (Thermo Fisher Scientific, Uppsala, Sweden). Positivity was defined as >10 arbitrary-units/ml. RF status was reported by the treating clinician.

### Sensitivity analysis

To assess the robustness of our findings and address potential selection bias introduced by patients in SRQb who were included at a later RA disease stage (i.e. implicitly conditioned on having failed treatment with MTX as DMARD monotherapy), we performed sensitivity analyses in a subcohort where we excluded all patients in SRQb whose blood samples for genotyping were taken >90 days after initiating MTX. Within the above subcohort, we re-evaluated the GWAS findings by testing all SNPs suggestively associated with either of the two treatment persistence outcomes. We considered a signal to be significant if it reached the nominal significance threshold (*P* < 0.05) after adjustment for multiple testing by Bonferroni correction of the number of tested SNPs. Furthermore, we performed additional estimation of heritability and evaluation of the RA PRS, as was done within the primary analysis.

### Supplementary analysis

In addition to the primary analysis cohort described above, we were able to incorporate a further 1326 additional samples included into SRQb after 2017, genotyped and imputed at a later time point independently from the samples in our primary analysis cohort. For these 1326 individuals to be included for subsequent analyses, the same criteria and definitions were applied as in the primary analysis cohort. These 1326 individuals were genotyped and imputed in a later round than those in the primary analysis cohort; we chose to pool the results (as opposed to pooling the data, analyzing the two datasets as one unique dataset). More specifically, we pooled the results from the GWAS and the PRS analysis, using METAL for the GWAS [32] and an inverse-variance weighted fixed effects model for the PRS associations. We chose to not perform the heritability estimation within this cohort of 1326 individuals due to the low sample size [33].

## Results

**Figure 1.**
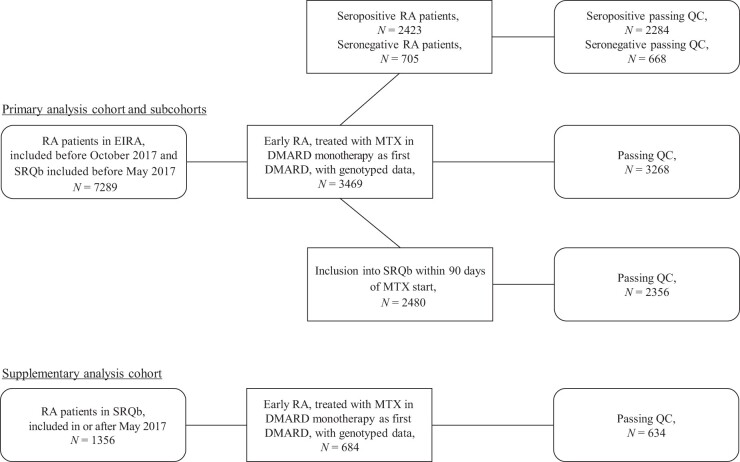
**Data extraction flowchart.** Flowchart visualization of the inclusion/exclusion criteria applied to obtain the primary analysis cohort, antibody-stratified subgroup, sensitivity analysis cohorts and the supplementary analysis cohort. QC: quality control; SRQb: Swedish Rheumatology Quality Register’s biobank; EIRA: Epidemiological Investigation of RA study

For our primary analysis cohort, we identified a total of 7289 RA patients from EIRA and SRQb, among whom 3469 were considered to be early-RA patients treated with MTX in DMARD monotherapy as their first-ever DMARD, and who had genome-wide SNP data available (Fig. 1). After QC, 3268 individuals and 5 978 812 SNPs remained for analysis (Supplementary Table S1, available at *Rheumatology* online). An additional 8 and 46 patients were excluded from the analyses at 1 and 3 years, respectively, due to death, migration or insufficient follow-up information to ascertain persistence.

### Occurrence of MTX DMARD monotherapy persistence

Among those remaining, 2112 (65%) were MTX treatment persistent at 1 year and 1412 (44%) were persistent at 3 years. Non-persistent patients were more often female, more often had seropositive RA and generally started treatment at an earlier age compared with persistent patients. The majority of non-persistent patients had added a non-MTX DMARD during the study period, with about 35% discontinuing MTX within 1 year. The registered reasons for discontinuation were similar within both outcomes, with discontinuation due to side effects being the largest group (Supplementary Table S2, available at *Rheumatology* online). Full cohort characteristics are presented in Table 1.

**Table 1.** Cohort characteristics

		Overall	**Persistence at 1** * * **year**		**Persistence at 3** * * **years**	
		*N* = 3268	Persistent *N* = 2112	Non-persistent *N* = 1148	Persistent *N* = 1412	Non-persistent *N* = 1810
**Demographics**						
	Female (%)	2283 (70%)	1443 (68%)	836 (73%)	946 (67%)	1311 (72%)
	Seropositive (%)	2305 (71%)	1467 (69%)	833 (73%)	960 (68%)	1315 (73%)
	Age (s.d.)	55 (13)	57 (13)	53 (14)	58 (13)	53 (14)
	Year of MTX start (IQR)	2009 (2005–13)	2009 (2005–13)	2009 (2006–13)	2009 (2006–13)	2009 (2005–13)
**Educational level**						
	<9 years (%)	743 (23%)	497 (24%)	245 (21%)	349 (25%)	380 (21%)
	9–12 years (%)	1544 (47%)	990 (47%)	552 (48%)	651 (46%)	877 (48%)
	≥12 years (%)	974 (30%)	623 (29%)	349 (30%)	410 (29%)	551 (30%)
**Rheumatic disease comorbidity index**						
	0 (%)	2587 (79%)	1651 (78%)	932 (81%)	1101 (78%)	1461 (81%)
	1 (%)	274 (8%)	180 (9%)	92 (8%)	114 (8%)	151 (8%)
	≥2 (%)	244 (7%)	162 (8%)	80 (7%)	110 (8%)	123 (7%)
**Baseline disease activity components**						
	SJC28 (s.d.)	8 (6)	8 (6)	9 (6)	8 (6)	8 (6)
	TJC28 (s.d.)	8 (6)	7 (6)	9 (6)	7 (6)	8 (6)
	CRP (s.d.)	22.85 (30.03)	20.29 (26.7)	27.53 (34.95)	20.33 (25.08)	24.71 (33.27)
	ESR (s.d.)	30.93 (22.64)	29.18 (21.58)	34.12 (24.2)	29.04 (20.98)	32.05 (23.7)
	Patient Global Health (s.d.)	48.98 (25.41)	46.34 (25.1)	53.88 (25.29)	44.75 (25.14)	52.1 (25.17)
	DAS28 (s.d.)	5.04 (1.32)	4.9 (1.3)	5.32 (1.3)	4.86 (1.29)	5.18 (1.32)
**MTX initiation characteristics**						
	Oral MTX (%)	2235 (68%)	1457 (69%)	775 (68%)	971 (69%)	1232 (68%)
	Folic acid supplementation (%)	2199 (67%)	1427 (68%)	768 (67%)	952 (67%)	1214 (67%)
	Prednisolone supplementation (%)	2800 (86%)	1766 (84%)	1030 (90%)	1158 (82%)	1611 (89%)
**Non-persistence characteristics**						
	Added a non-MTX DMARD (%)			994 (87%)		1568 (86%)
	Discontinued MTX (%)			438 (38%)		889 (50%)

Descriptive characteristics of the primary analysis cohort at the time of MTX initiation, consisting of Swedish early-RA patients treated with MTX in DMARD-monotherapy as their first prescribed treatment for RA, stratified by treatment persistence status. DAS28: DAS based on 28 joint counts; SJC28: swollen joint count in 28 joints; TJC28: tender joint count in 28 joints.

### Genome-wide assessment of individual SNPs as predictors of MTX DMARD monotherapy persistence

No individual SNP reached the genome-wide significance threshold (*P* < 5 × 10^–^^8^) for either of the two outcomes. For persistence at 1 year, 153 SNPs passed the suggestive threshold (*P* < 5 × 10^–^^5^). This corresponded to 42 regional clusters across 18 different chromosomes (Supplementary Fig. S1, Supplementary Table S3, available at *Rheumatology* online), where a regional cluster was defined as all SNPs within 200 kb of the lead SNP. For persistence at 3 years, 322 SNPs surpassed the suggestive threshold, corresponding to 47 regional clusters of SNPs across 19 different chromosomes (Supplementary Fig. S2, Supplementary Table S4, available at *Rheumatology* online).

### An RA polygenic risk score as predictor of MTX DMARD monotherapy persistence

After QC and filtration, 2 682 494 of the RA GWAS SNPs [24] remained for estimation of PRS weights. Of these, 1 718 182 overlapped between EIRA and SRQb. The PRS was not significantly associated with MTX treatment persistence at 1 year (RR = 0.98, 95% CI 0.96–1.01) or 3 years (RR = 0.96 95% CI 0.93–1.00). These results remained after adjusting for covariates, both for MTX treatment persistence at 1 year (RR = 0.99, 95% CI 0.96–1.01) and 3 years (RR = 0.97, 95% CI 0.93–1.01). Estimates were similar when contrasting patients across PRS quintiles, though no statistically significant associations were observed (Supplementary Table S5, available at *Rheumatology* online).

### SNP-based heritability of MTX DMARD monotherapy persistence

For persistence at 1 year, the crude SNP-based heritability (*h*^2^) was estimated at *h*^2^ = 0.45 (95% CI 0.15–0.75). In the adjusted model, the point-estimate was similar (*h*^2^ = 0.46, 95% CI 0.15–0.78). Similarly, the crude SNP-based heritability of persistence at 3 years was estimated at *h*^2^ = 0.14 (95% CI 0*–0.40), with an adjusted estimate of *h*^2^ = 0.03 (95% CI 0*–0.33), the asterisk denoting rounding to zero.

### Seropositive and seronegative subcohort analysis

Of the 3469 patients available from the primary analysis cohort, 2423 (70%) were categorized as seropositive and 705 (20%) as seronegative. A total of 332 patients (10%) had incomplete data on autoantibody status and were excluded from the stratified analyses. After QC, 2284 seropositive and 668 seronegative patients, both with data on ∼6 million SNPs, remained for subcohort analyses (Supplementary Table S1, available at *Rheumatology* online). In seropositive patients, 5 and 31 individuals had missing information on persistence at 1 and 3 years, respectively; in seronegative patients, 3 and 10 had missing information on MTX treatment persistence at 1 and 3 years, respectively. These individuals were excluded from their respective analyses.

Among seropositive patients, 1450 (64%) and 953 (43%) were MTX treatment persistent at 1 and 3 years, respectively; among seronegative patients, 430 (63%) and 303 (46%) were persistent at 1 and 3 years, respectively. The demographic characteristics were generally comparable with those observed within the primary analysis cohort, although there were slightly more male patients among those with seronegative RA. Furthermore, there were slightly more patients discontinuing MTX among the non-persistent seronegative patients (Table 2). Otherwise, the reason for discontinuation was comparable across outcomes and strata (Supplementary Table S2, available at *Rheumatology* online).

**Table 2.** Cohort characteristics and results in seropositive and seronegative subgroups

		SEROPOSITIVE RA				SERONEGATIVE RA			
		*N* = 2284				*N* = 668			
		Persistence at 1 year		Persistence at 3 years		Persistence at 1 year		Persistence at 3 years	
		Persistent	Non-persistent	Persistent	Non-persistent	Persistent	Non-persistent	Persistent	Non-persistent
		*N* = 1450	*N* = 829	*N* = 953	*N* = 1300	*N* = 430	*N* = 235	*N* = 303	*N* = 355
**Demographics**									
	Female (%)	995 (69%)	610 (74%)	639 (67%)	951 (73%)	283 (66%)	160 (68%)	198 (65%)	243 (68%)
	Age (s.d.)	56 (13)	53 (14)	57 (13)	53 (13)	59 (13)	53 (14)	59 (12)	54 (14)
	Year of MTX start (IQR)	2009 (05-12)	2008 (05-12)	2009 (05-12)	2008 (05-13)	2009 (05-13)	2008 (05-13)	2008 (05-13)	2008 (04-13)
**Educational level**									
	<9 year (%)	326 (22%)	177 (21%)	217 (23%)	274 (21%)	130 (30%)	57 (24%)	102 (34%)	84 (24%)
	9–12 years (%)	701 (48%)	403 (49%)	459 (48%)	636 (49%)	187 (43%)	114 (49%)	121 (40%)	177 (50%)
	≥12 years (%)	421 (29%)	248 (30%)	275 (29%)	389 (30%)	113 (26%)	64 (27%)	80 (26%)	94 (26%)
**Rheumatic disease comorbidity index**									
	0 (%)	1137 (78%)	666 (80%)	745 (78%)	1045 (80%)	315 (73%)	192 (82%)	221 (73%)	282 (79%)
	1 (%)	119 (8%)	70 (8%)	74 (8%)	111 (9%)	49 (11%)	18 (8%)	34 (11%)	30 (8%)
	≥2 (%)	102 (7%)	53 (6%)	67 (7%)	80 (6%)	40 (9%)	20 (9%)	29 (10%)	31 (9%)
**Baseline disease activity components**									
	SJC28 (s.d.)	8 (5)	9 (6)	8 (6)	8 (5)	9 (6)	9 (5)	9 (6)	9 (6)
	TJC28 (s.d.)	7 (6)	8 (6)	7 (6)	8 (6)	7 (6)	9 (6)	7 (5)	9 (7)
	CRP (s.d.)	19.38 (25.39)	27.76 (35.59)	19.27 (24.17)	24.63 (32.95)	23.11 (27.9)	28.13 (34.7)	23.48 (27.59)	25.93 (32.97)
	ESR (s.d.)	28.79 (21.42)	34.46 (24.47)	28.49 (20.72)	32.25 (23.86)	28.92 (22.42)	32.22 (22.37)	29.08 (21.74)	30.68 (22.95)
	Patient Global Health (s.d.)	46.14 (24.95)	54.05 (25.38)	44.43 (24.99)	52.23 (25.14)	47.48 (24.65)	52.74 (25.84)	45.84 (24.44)	52.25 (25.53)
	DAS28 (s.d.)	4.85 (1.29)	5.29 (1.31)	4.81 (1.28)	5.15 (1.32)	4.92 (1.31)	5.38 (1.25)	4.87 (1.26)	5.24 (1.33)
**MTX initiation characteristics**									
	Oral MTX (%)	959 (66%)	536 (65%)	626 (66%)	851 (65%)	278 (65%)	164 (70%)	198 (65%)	239 (67%)
	Folic acid supplementation (%)	934 (64%)	527 (64%)	611 (64%)	832 (64%)	275 (64%)	166 (71%)	194 (64%)	242 (68%)
	Prednisolone supplementation (%)	1208 (83%)	744 (90%)	775 (81%)	1162 (89%)	347 (81%)	206 (88%)	242 (80%)	305 (86%)
**Liability-scale SNP-heritability**									
	Crude *h*^2^ (s.d.)	0.46 (0.21)		0.20 (0.19)		0.34 (0.72)		0.00 (0.70)	
	Adjusted^a^*h*^2^ (s.d.)	0.36 (0.23)		0.00 (0.22)		0.25 (0.76)		0.00 (0.73)	
**Polygenic risk score, RR**									
	Crude RR (95% CI)	0.98 (0.95, 1.01)		0.96 (0.91, 1.00)		1.02 (0.96, 1.08)		1.01 (0.93, 1.09)	
	Adjusted^a^ RR (95% CI)	0.99 (0.96, 1.02)		0.97 (0.92, 1.01)		1.01 (0.96, 1.06)		1.01 (0.93, 1.10)	

Descriptive characteristics at the time of MTX initiation, liability-scale heritability estimates and RRs quantifying the effect of the RA PRS on the persistence outcomes, in strata of seropositive and seronegative early-RA patients, treated with MTX in DMARD monotherapy as their first ordinated treatment.

a Adjusted for age, sex, and genetic ancestry per principal components.

DAS28: DAS based on 28 joint counts; *h*^2^: heritability; RR: risk ratio; SJC28: swollen joint count in 28 joints; TJC28: tender joint count in 28 joints.

In the primary analysis cohort, no SNP had a genome-wide significant association with either persistence trait, neither within the seropositive nor the seronegative subgroup. Among the seropositive patients, 269 SNPs showed suggestive associations (*P* < 5 × 10^–^^5^) with MTX treatment persistence at 1 year, clustering over 52 regions (Supplementary Fig. S3a, available at *Rheumatology* online), and a set of 259 SNPs showed suggestive associations with MTX treatment persistence at 3 years, clustering over 45 regions (Supplementary Fig. S3b, available at *Rheumatology* online). Similarly, among seronegative patients, we identified 352 SNPs suggestively associated with persistence at 1 year, clustering over 42 regions (Supplementary Fig. S3c, available at *Rheumatology* online), with a set of 378 SNPs suggestively associated with persistence at 3 years, clustering over 33 regions (Supplementary Fig. S3d, available at *Rheumatology* online).

Among seropositive patients, PRS associations were comparable with those observed within the primary analysis cohort, with a slight increase in effect size during categorical modelling across quintiles (Supplementary Table S5, available at *Rheumatology* online). Heritability estimates were comparable with those from primary analyses, although the point-estimates were slightly attenuated, with wider CIs (Table 2). In seronegative patients, PRS effects were further attenuated towards the null (Table 2), with no discernable trend when comparing patients across PRS quintiles (Supplementary Table S5, available at *Rheumatology* online). Heritability estimates were further attenuated, with substantial standard errors (Table 2).

### Sensitivity analysis

After removing all patients who were included in SRQb >90 days after start of MTX, or who failed to meet the QC criteria, 2356 individuals and 5 984 513 SNPs remained for analysis (Supplementary Table S1, available at *Rheumatology* online). Of those remaining, 8 and 39 were censored at 1 and 3 years, and excluded from their respective analyses. The demographic characteristics were comparable with those observed in the primary cohort (Supplementary Table S6, available at *Rheumatology* online).

Among the SNPs showing a suggestive association (*P* < 5 × 10^–^^5^) with MTX treatment persistence at 1 year, 70 (45%) remained significant, passing the Bonferroni-corrected significance threshold. Similarly, among those surpassing a suggestive association with persistence at 3 years, 133 (41%) remained significant. The heritability point-estimates were comparable with those obtained for the primary analysis cohort, though the CIs were wider (Supplementary Table S6, available at *Rheumatology* online). The PRS point-estimates were similar to those observed for the primary analysis cohort, though ultimately non-significant in both univariate and adjusted models, with respect to both outcomes (Supplementary Table S6, available at *Rheumatology* online).

### Supplementary analysis

For the supplementary analysis cohort, we identified a total of 1326 RA patients included in SRQb after 2017, among which 694 individuals met the inclusion criteria. Of these, 634 individuals and around 8 million SNPs remained for analysis after quality control (Supplementary Table S1, available at *Rheumatology* online). Cohort demographics were generally comparable with those observed within the primary analysis cohort, although the patients were slightly older and had slightly lower baseline disease activity (Supplementary Table S7, available at *Rheumatology* online). The pooled GWAS results (based on the 3268 individuals from the primary analysis cohort and the 634 individuals from the supplementary analysis cohort) did not differ appreciably from those observed for the primary analysis cohort, with no SNPs reaching genome-wide significance alongside 331 and 308 suggestive associations for MTX treatment persistence at 1 and 3 years, respectively. Pooled RRs from the PRS analysis were similar, with slightly tighter CIs, though similarly non-significant, for both persistence at 1 (RR = 0.99, 95% CI 0.97–1.00) and 3 years (RR = 0.97 95% CI 0.94–1.00).

## Discussion

We present the largest GWAS on an MTX treatment outcome to date, and the first on MTX treatment persistence in DMARD monotherapy in early RA. Despite a moderate sample size and only including early, DMARD-naïve RA patients, no SNPs were associated with either of the two outcomes at a genome-wide significant level. Nevertheless, SNP-based heritability point-estimates were modest and PRS associations robust, implying that MTX treatment persistence has a genetic component, even if its exact nature and size was difficult to ascertain.

Previous GWASs on MTX treatment outcomes have not yielded validated genome-wide significant SNP associations [34–37]. The findings have also been discordant, with studies failing to replicate suggestive signals from preceding studies [36]. One explanation for such discrepancies could be the underlying genetic architecture attributed to the various treatment outcomes. Exemplifying the genetic distinctness of treatment phenotypes, a previous study found substantial differences in heritability across different treatment outcomes within RA patients, with only low to modest phenotypic correlation between them [38]. Previous studies have primarily focused on primary response, whereas we instead investigated the more heterogeneous outcome of MTX treatment persistence in DMARD monotherapy. Nevertheless, the decision to stop treatment with MTX in DMARD monotherapy is likely to be guided by observed disease activity, implying high phenotypic correlation with common effectiveness measures such as DAS28. Additionally, our MTX treatment persistence outcome has the benefit of capturing poor tolerance, in addition to long-term treatment outcomes such as slower drug response. Nevertheless, failure to replicate signals could also be explained by differences in cohort characteristics. This is relevant within the current context, because other GWAS on MTX treatment outcomes have had major differences when patient ethnicity and disease duration differs [35], and have occasionally been performed in non-RA patients [34, 37] making comparisons challenging between different studies.

Our primary analysis identified a small, albeit non-significant, inverse association between the PRS for RA risk and MTX treatment persistence in DMARD monotherapy, indicating that RA genetics may have some predictive capacity for whether a patient remains on MTX treatment in DMARD monotherapy. While such an effect was small, one potential interpretation of these results suggests that individuals genetically predisposed towards RA are more likely to develop an aggressive disease phenotype, thus requiring early escalation of therapy. Furthermore, subgroup analyses stratified according to autoantibody status indicated a larger effect of the PRS on MTX treatment persistence within seropositive patients, and an attenuated effect in seronegative patients. These results are perhaps not surprising and could be explained by differences in genetic architecture, as seropositive RA is reported to be more heritable [39], has stronger associations at the *HLA* locus [40] and has a larger set of established genetic associations than its seronegative counterpart [41].

In general, SNP-based estimates of heritability tend to underestimate true narrow-sense heritability, whereas estimates from family-based studies can overestimate the same quantity [29, 42, 43]. However, when comparing our estimates with those obtained from our previous family-based study within the same source population, the results were discordant. Whereas our current study indicated greater heritability for MTX treatment persistence at 1 year compared with persistence at 3 years, the family-based estimates suggested the opposite, with a higher heritability at 3 years ($h_{\mathrm{FAM}}^{2}\;$= 0.58, 95% CI 0.27–0.89) than at 1 year ($h_{\mathrm{FAM}}^{2}$ = 0.08, 95% CI 0–0.43) [11]. One reasonable source of this discrepancy is the high RA-population heterogeneity, reflected in both studies by the wide CIs including the majority of possible values. Considerably larger sample sizes would be required in order to obtain precise heritability estimates.

Our study had some limitations. First, estimation of SNP-based heritability was performed through GREML [28, 29]. While this approach is well established, it has been shown to perform worse than certain model extensions when relying on data including imputed variants, as was the case here [44]. However, alternative approaches require substantial samples to perform efficiently (a sample size of >20 000 was recommended in order to obtain standard errors of <0.05); we decided to opt for the standard GREML approach, as precision was already an issue. Second, selection bias was a potential concern, as a subset of SRQb participants had been sampled at a later stage of their disease course and were implicitly conditioned on having failed treatment with MTX in DMARD monotherapy. To quantify their influence on our results, we performed sensitivity analyses following exclusion of all SRQb participants with blood samples taken >90 days after MTX initiation. The GWAS results based on this failed to identify the majority of suggested associations observed within the primary analysis, despite the more liberal significance threshold. While this may raise concern about the validity of our GWAS, it should be noted that the results from the heritability analysis and the PRS analysis, within the same subcohort, were highly concordant with the results from the primary analysis. Third, while results from the PRS analysis were robust throughout the study, performance may have been improved by additionally including SNPs in perfect LD, which were here filtered out to reduce computational burden. Furthermore, the predictive performance of LDpred2 has been shown to decrease when not using per-SNP sample sizes [45], a quantity unavailable for our data, and this could have had an effect on our PRS calculation.

In the context of existing literature on the pharmacogenetics of MTX treatment response, the main strengths of our study lie in the size and homogeneity of the cohort. Focusing on early DMARD-naïve RA reduces the risk of selection bias introduced by the inclusion of patients already tolerating the drug. Furthermore, the outcome of MTX treatment persistence functions as a clinically relevant proxy for the combined impact of effectiveness and tolerance, which are often difficult to fully separate, while simultaneously suffering less from missingness than commonly studied measures such as DAS28, which require consecutive clinical examination.

In conclusion, we did not find any genome-wide significant associations of SNPs with MTX persistence. Nevertheless, heritability estimates indicated a consistent, albeit modest, genetic contribution to the phenotype. This, coupled with the widespread nature of suggestively associated loci, further indicates a polygenic architecture. The collective evidence points towards the unlikeliness of individual genetic variants as clinically useful biomarkers of treatment response. However, our findings suggest that aggregation of genetic data may still, especially in combination with additional clinical and lifestyle data, play a role in predicting RA treatment outcome and should be further investigated in multiple patient groups.

## Supplementary Material

kead301_Supplementary_Data

## Data Availability

For reasons related to the ethical and legal permits surrounding the individual study data, these cannot be shared publicly; requests pertaining to data access can be directed to the EIRA secretariat (http://www.eirasweden.se) and the SRQ biobank (https://srq.nu/en/). All sets of GWAS summary statistics are made available via the GWAS Catalog and first author's GitHub page.
