# Supplementary material for: Genome-wide investigation of persistence with methotrexate treatment in early rheumatoid arthritis
Source: Rheumatology (Oxford). 2023 Jun 16;63(5):1221–9. doi: 10.1093/rheumatology/kead301 (PMC11065441; doi:10.1093/rheumatology/kead301)
Supplement: kead301_Supplementary_Data [file kead301_supplementary_data.docx]

**SUPPLEMENTARY MATERIAL FOR “GENOME-WIDE INVESTIGATION OF PERSISTENCE TO TREATMENT WITH METHOTREXATE IN EARLY RHEUMATOID ARTHRITIS”**

**Anton Öberg Sysojev^1^, Saedis Saevarsdottir^1,2,3^, Lina Marcela Diaz-Gallo^4,5^, Gilad N. Silberberg^4,5^, Lars Alfredsson^6^, Lars Klareskog^4,5^, Eva Baecklund^7^, Lena Björkman^8^, Alf Kastbom^9^, Solbritt Rantapää-Dahlqvist^10^, Carl Turesson^11^, Ingileif Jonsdottir^2,3^, Kari Stefansson^2,3^, Thomas Frisell^1^, Leonid Padyukov^4,5^, Johan Askling^1,7^, Helga Westerlind^1^**

^1^Clinical Epidemiology Division, Department of Medicine Solna, Karolinska Institute, Stockholm, Sweden.

^2^Faculty of Medicine, School of Health Sciences, University of Iceland, Reykjavik, Iceland.

^3^deCODE genetics Inc, Reykjavik, Iceland.

^4^Division of Rheumatology, Department of Medicine Solna, Karolinska Institute and Karolinska University Hospital, Stockholm, Sweden.

^5^Center for Molecular Medicine, Department of Medicine Solna, Karolinska Institute, Stockholm, Sweden.

^6^Institute of Environmental Medicine (IMM), Karolinska Institute, Stockholm, Sweden.

^7^Department of Medical Sciences, Rheumatology, Uppsala University, Uppsala, Sweden.

^8^Department of Rheumatology and Inflammation Research, University of Göteborg, Göteborg, Sweden.

^9^Department of Biomedical and Clinical Sciences, Linköping University, Linköping, Sweden.

^10^Department of Public Health and Clinical Medicine, Rheumatology, Umeå University, Umeå, Sweden.

^11^Department of Clinical Sciences, Malmö, Lund University, Malmö, Sweden.

^12^Rheumatology, Theme Inflammation and Ageing, Karolinska University Hospital Solna, Stockholm, Sweden

Corresponding author: Anton Öberg Sysojev; Clinical Epidemiology Division, Eugeniahemmet, T2, Karolinska Universitetssjukhuset, Solna 171 76, Stockholm, Sweden; anton.oberg.sysojev@ki.se; +46 (0)739651247; 0000-0003-3221-7979.

**SUPPLEMENTARY NOTE**

*Genotyping details*

For the rheumatoid arthritis (RA) patients obtained from the epidemiological investigation of RA (EIRA) study, blood samples were obtained from local health care units at the time of the first visit [1]. Samples were collected and stored at the Centre for Molecular Medicine, Karolinska Institutet, Stockholm, Sweden, prior to DNA extraction, storage conducted in -80°C freezers. Participants in the Swedish Rheumatology Quality register (SRQ) were asked for their participation in the SRQ biobank (SRQb). Blood samples were taken from consenting patients at their local rheumatology clinic and sent to Karolinska Institutet, Stockholm, Sweden, for subsequent storage and DNA extraction [2]. Genotyping of both samples was performed at deCODE, Iceland, on the Illumina Infinium Global Screening Array.

*Imputation details*

Genotyped data on the EIRA and SRQb participants was combined and filtered for quality control of genotyped variants prior to imputation. In particular, quality control steps included removing individuals/markers that had: (i) individual call rate < 0.95; (ii) SNP call rate < 0.95; (iii) SNP minor allele frequency < 0.01; (iv): SNP deviation from Hardy-Weinberg equilibrium (p < 1e-4). Subsequent imputation was done against the European subset of the 1000 Genomes Phase 3 [3] reference panel, using IMPUTE2 (v2.3.2) (<https://mathgen.stats.ox.ac.uk/impute/impute_v2.html>) [4].

*Quality control details*

Stringent quality control was performed in both individuals and genetic variants. Samples were excluded based on the following criteria: (i) SNP imputation R^2^ < 0.7; (ii) individual reported sex not matching inferred sex, or ambiguous inferred sex (where X chromosome homozygosity rate > 0.8 was interpreted as male sex and < 0.2 as female sex); (iii) individual call rate < 0.95; (iv) SNP call rate < 0.95; (v) SNP minor allele frequency < 0.01; (vi) SNP exhibiting significant deviation from Hardy-Weinberg equilibrium (p < 1e-6 in the full cohort); (vii) individual degree of pairwise relatedness > 0.125; (viii) individual non-homogeneous genetic ancestry (individuals more than six standard deviations from the principal component mean on any of the ten first principal components, passing over five iterations). Here, the final two quality control steps, (vii) and (viii), were performed in a subset of SNPs where high linkage disequilibrium (LD) SNPs had been withheld. In particular, SNPs in 24 regions of high LD [5], and SNPs identified by stringent LD pruning (employing an r^2^ threshold of 0.2) were removed prior to these steps, and subsequently returned after completion of quality control, i.e. withheld for the purpose of performing quality control steps vii-viii to avoid biases from highly correlated SNPs. All quality control steps were carried out in PLINK (v1.90b) [6, 7]. Results of the quality control procedure are presented in Table S1.

An identical quality control procedure was carried out on the 1000 Genomes Phase 3 [3] European subpopulation data, used as an LD reference panel for LDpred2. In addition to the above, SNPs overlapping the post-QC reference panel data and the RA GWAS summary statistic data [8] were further filtered to exclude those not compliant with LDpred2 assumptions [9]. In particular, this meant verifying that

$$sd\left( G_{j} \right)\approx\frac{2}{se\left( \hat{\gamma}_{j} \right)\sqrt{n_{eff}}}, j=1, \ldots, M,$$

where $G_{j}$ denotes the observed genotypes of the *j*’th SNP in the LD reference panel data, $se\left( \hat{\gamma}_{j} \right)$ is the estimated standard error of the *j*’th SNP in the GWAS summary statistic data and $n_{eff}= \frac{4}{1/n_{case}+1/n_{ctrl}}$ is the effective sample size.

**SUPPLEMENTARY FIGURES**


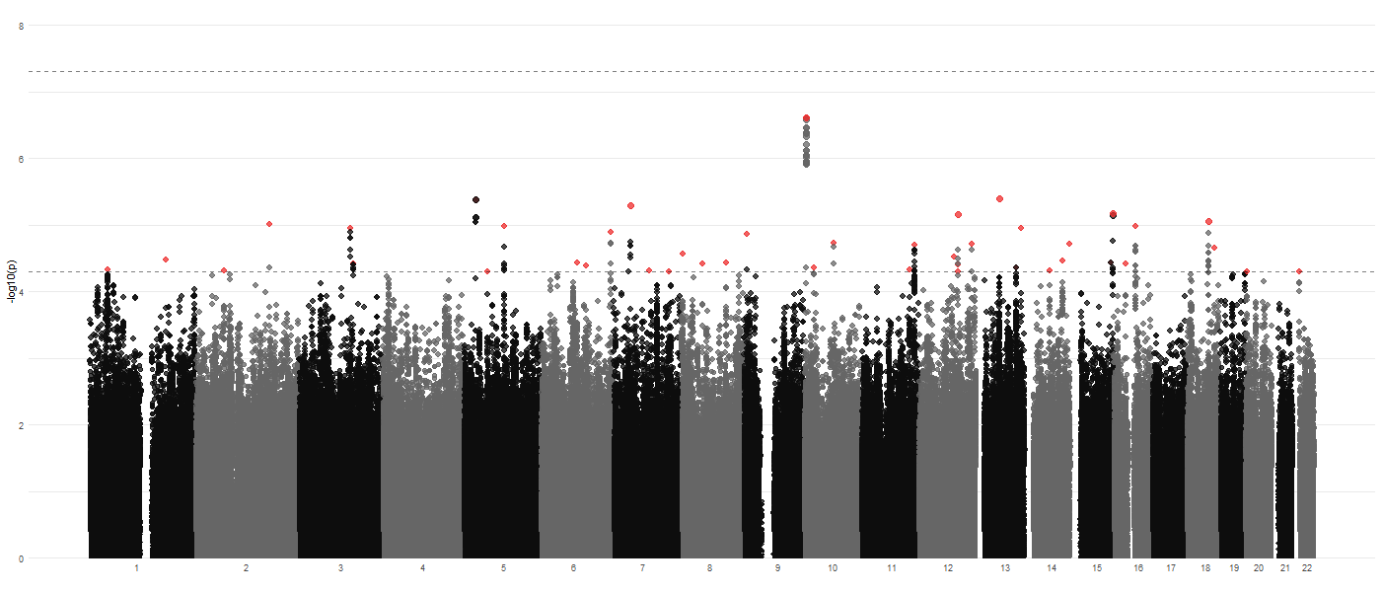


**Supplementary Figure S1: Manhattan plot of results from the GWAS on persistence to methotrexate at one year within the primary analysis cohort.** *The dashed line indicates the genome-wide significance threshold (p < 5e-8), the dotted line indicates the suggestive threshold (p < 5e-5). Points highlighted in red indicate the lead SNP of distinct regional clusters.*


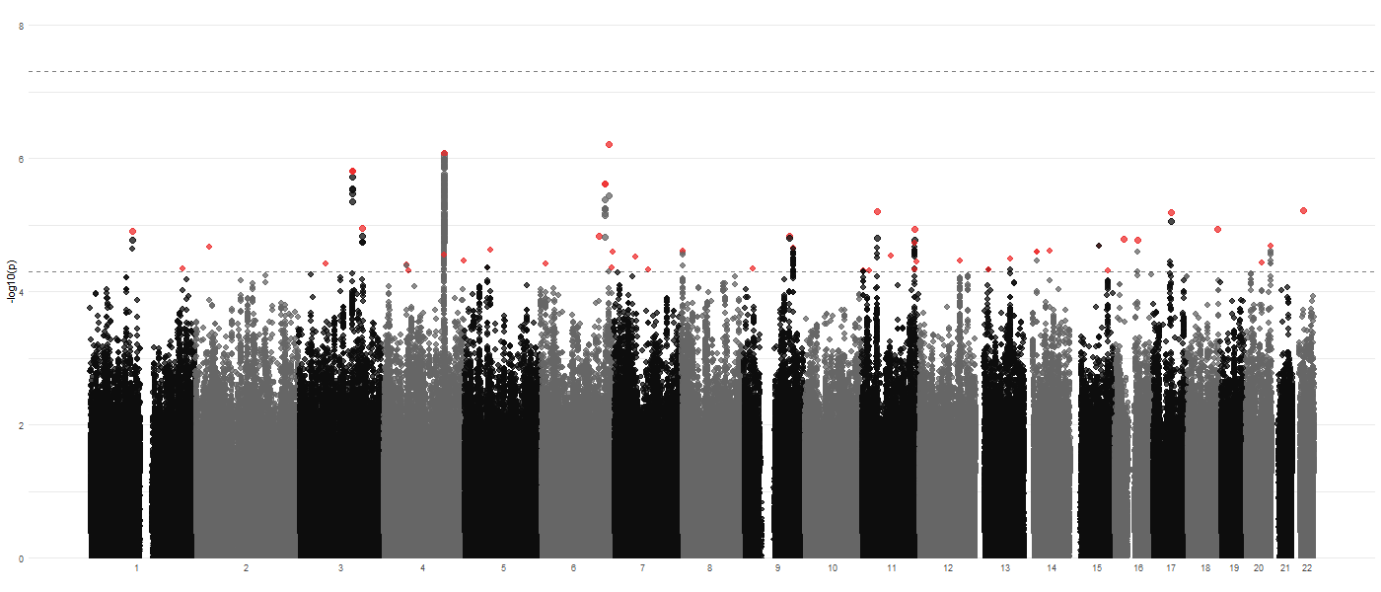


**Supplementary Figure S2: Manhattan plot of results from the GWAS on persistence to methotrexate at three years within the primary analysis cohort.** *The dashed line indicates the genome-wide significance threshold (p < 5e-8), the dotted line indicates the suggestive threshold (p < 5e-5). Points highlighted in red indicate the lead SNP of distinct regional clusters.*


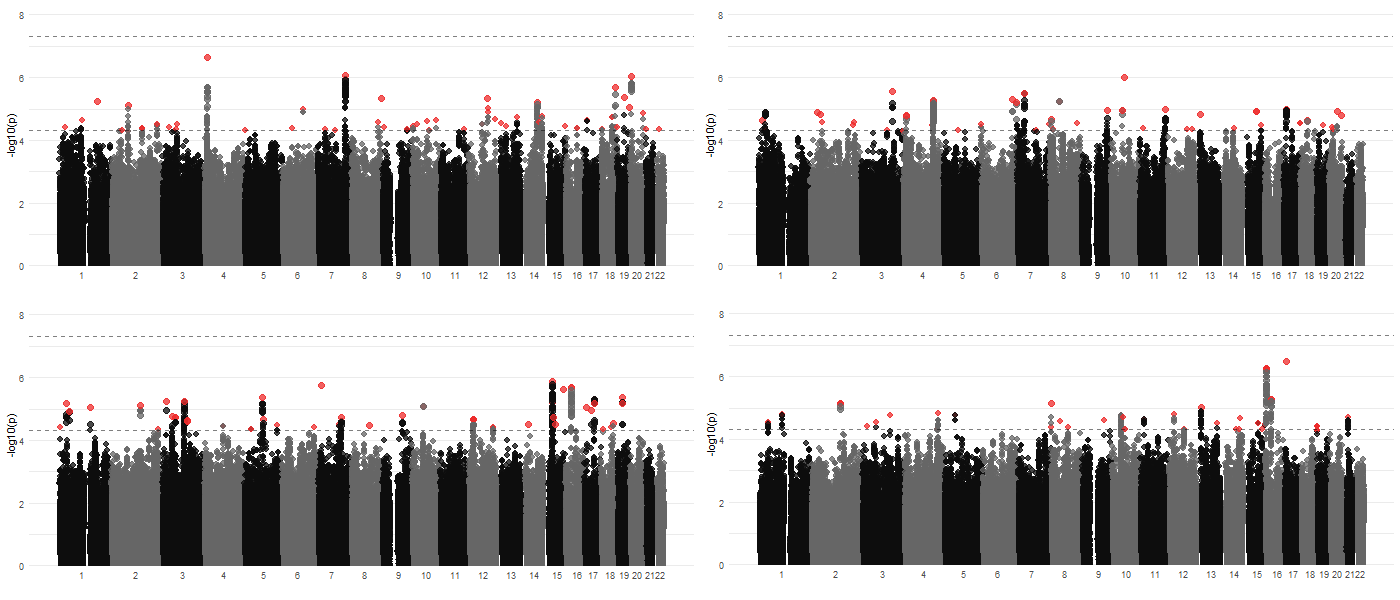


**Supplementary Figure S3: Manhattan plots of results from the GWASs on persistence to methotrexate at one and three years, in sub-cohorts of seropositive and seronegative RA.** *The dashed line indicates the genome-wide significance threshold (p < 5e-8), the dotted line indicates the suggestive threshold (p < 5e-5). Points highlighted in red indicate the lead SNP of distinct regional clusters. Top row illustrates results from the seropositive analysis with the outcome of persistence at one year on the left and persistence at three years on the right. The bottom row analogously illustrates results from the seronegative analysis.*

**SUPPLEMENTARY TABLES**

**Supplementary Table S1: Quality control procedure.** *Summary of the samples and SNPs removed during the quality control procedure for the primary analysis cohort containing overall RA patients, the seropositive and -negative sub-cohorts, the sensitivity analysis sub-cohort as well as the supplementary analysis cohort.*

|  | OVERALL RA | SERO+ RA | SERO- RA | SENSITIVITY | SUPPLEMENTARY |
| --- | --- | --- | --- | --- | --- |
| **TOTAL PATIENTS GENOTYPED** | **3469** | **2423** | **705** | **3469** | **684** |
| Reported sex  matching inferred sex,  and non-ambiguous inferred sex | 3456 | 2412 | 703 | 3456 | 684 |
| Call rate ≥ 0.95 | 3351 | 2334 | 685 | 3351 | 681 |
| Pairwise relatedness ≤ 0.125 | 3310 | 2305 | 684 | 3310 | 663 |
| Comparable genetic ancestry | 3268 | 2284 | 668 | 3268 | 634 |
| **TOTAL PATIENTS REMAINING** | **3268** | **2284** | **668** | **3268** | **634** |
|  | | | | |  |
| **TOTAL SNPS GENOTYPED AND IMPUTED** | **48,434,495** | | | | **48,434,495** |
| Imputation quality ≥ 0.70 | 13,614,054 | | | | 13,614,054 |
| Call rate ≥ 0.95 | 10,831,799 | 10,829,768 | 10,831,464 | 10,836,847 | 12,709,347 |
| Minor allele frequency ≥ 0.01 | 5,980,462 | 5,976,059 | 5,969,705 | 5,985,227 | 7,758,107 |
| Compliance with Hardy-Weinberg equilibrium (p ≥ 1e-6) | 5,978,812 | 5,975,193 | 5,969,557 | 5,984,513 | 7,757.556 |
| **TOTAL SNPS REMAINING** | **5,978,812** | **5,975,193** | **5,969,557** | **5,984,513** | **7,757.556** |

**Supplementary Table S2: Reason for MTX discontinuation.** *Recorded reason for discontinuation of MTX, per the Swedish Rheumatology Quality register, in the primary analysis cohort as well as the seropositive and -negative subcohorts.*

| **REASON FOR DISCONTINUING MTX AMONG NON-PERSISTENT PATIENTS** | | | | | | |
| --- | --- | --- | --- | --- | --- | --- |
|  | **Discontinued MTX within one year** | | | **Discontinued MTX within three years** | | |
|  | **Overall RA** | **Seropositive RA** | **Seronegative RA** | **Overall RA** | **Seropositive RA** | **Seronegative RA** |
| **N** | 438 | 310 | 86 | 889 | 611 | 191 |
| **Side effects (%)** | 133 (0.3) | 95 (0.31) | 17 (0.2) | 234 (0.26) | 165 (0.27) | 38 (0.2) |
| **Inadequate effect (%)** | 9 (0.02) | 5 (0.02) | 2 (0.02) | 31 (0.03) | 17 (0.03) | 10 (0.05) |
| **Remission (%)** | 10 (0.02) | 6 (0.02) | 4 (0.05) | 45 (0.05) | 21 (0.03) | 15 (0.08) |
| **Other (%)** | 46 (0.11) | 25 (0.08) | 13 (0.15) | 128 (0.14) | 82 (0.13) | 27 (0.14) |
| **Unknown^**^ (%)** | 240 (0.55) | 179 (0.58) | 50 (0.58) | 451 (0.51) | 326 (0.53) | 101 (0.53) |
| *: The category of ‘Other’ is a grouping of the five categories of ‘Switch from original to biosimilar’, ‘Patient withdrawn from SRQ’, ‘Planned treatment’, ‘Comorbidity or surgery’, ‘Pregnancy or planned pregnancy’ and ‘Patient decision’, grouped here due to very low counts within the individual categories.  **: The category of ‘Unknown’ is a grouping of the two categories of ‘Other or unknown reason’ and ‘Unknown reason’. | | | | | | |

**Supplementary Table S3: GWAS top SNPs for persistence to methotrexate at one year within the primary analysis cohort.** *Summary of SNPs associated with persistence at one year at a level of p < 5e-5, grouped according to their regional clusters. A regional cluster is defined as all SNPs reaching p < 5e-5 within 200kb of the lead SNP. Bolded SNPs indicate the lead SNP, and p_pool_ denotes p-values in the pooled meta-analysis. Reported positions are per hg19.*

| Region | SNP | Chromosome | Position | Effect allele | N | OR | 95% CI | P | P_pool_ |
| --- | --- | --- | --- | --- | --- | --- | --- | --- | --- |
| **1** | **rs2152314** | **1** | **42946462** | **T** | **3123** | **0.79** | **0.70-0.88** | **4.70e-05** | 1.07e-03 |
| **2** | **rs12079690** | **1** | **180568720** | **C** | **3222** | **0.74** | **0.64-0.85** | **3.32e-05** | 1.25e-03 |
| **3** | **2:67236289:T:C** | **2** | **67236289** | **T** | **3205** | **1.34** | **1.17-1.55** | **4.86e-05** | 5.79e-06 |
| **4** | **2:173254244:C:T** | **2** | **173254244** | **T** | **3227** | **2.6** | **1.70-3.98** | **9.84e-06** | 3.37e-05 |
| 4 | rs201590863 | 2 | 173292287 | TA | 3173 | 3.63 | 1.96-6.73 | 4.38e-05 | 1.13e-04 |
| **5** | **rs62269244** | **3** | **121805228** | **T** | **3244** | **0.67** | **0.56-0.80** | **1.11e-05** | 3.08e-05 |
| 5 | rs13090103 | 3 | 121811585 | A | 3254 | 0.7 | 0.60-0.83 | 2.37e-05 | 3.69e-05 |
| 5 | rs13064913 | 3 | 121814511 | G | 3243 | 0.71 | 0.60-0.83 | 3.03e-05 | 4.98e-05 |
| 5 | rs34652460 | 3 | 121815618 | G | 3254 | 0.7 | 0.60-0.82 | 1.55e-05 | 3.17e-05 |
| 5 | rs62269247 | 3 | 121818962 | A | 3254 | 0.7 | 0.60-0.82 | 1.55e-05 | 3.40e-05 |
| 5 | rs3792285 | 3 | 121819586 | A | 3255 | 0.7 | 0.59-0.82 | 1.27e-05 | 3.14e-05 |
| **6** | **rs73865022** | **3** | **128172207** | **T** | **3255** | **0.55** | **0.42-0.73** | **3.82e-05** | 4.03e-05 |
| 6 | rs76123284 | 3 | 128172721 | C | 3255 | 0.55 | 0.42-0.73 | 3.82e-05 | 4.03e-05 |
| 6 | rs58470498 | 3 | 128173413 | T | 3254 | 0.55 | 0.42-0.73 | 3.89e-05 | 3.58e-05 |
| 6 | rs57569230 | 3 | 128173536 | C | 3254 | 0.55 | 0.42-0.73 | 3.89e-05 | 3.58e-05 |
| 6 | rs57936870 | 3 | 128174131 | A | 3258 | 0.56 | 0.42-0.74 | 4.71e-05 | 4.40e-05 |
| 6 | rs57029453 | 3 | 128174311 | T | 3258 | 0.56 | 0.42-0.74 | 4.71e-05 | 4.40e-05 |
| 6 | rs55999119 | 3 | 128174635 | G | 3259 | 0.56 | 0.42-0.74 | 4.63e-05 | 4.29e-05 |
| 6 | rs73865024 | 3 | 128175395 | T | 3255 | 0.56 | 0.42-0.74 | 4.44e-05 | 2.60e-05 |
| 7 | rs36052500 | 5 | 26876097 | T | 3215 | 0.49 | 0.37-0.67 | 4.10e-06 | 2.37e-06 |
| 7 | rs12188963 | 5 | 26876499 | G | 3214 | 0.5 | 0.37-0.67 | 4.19e-06 | 2.41e-06 |
| **7** | **rs12188958** | **5** | **26906566** | **C** | **3226** | **0.51** | **0.38-0.69** | **9.23e-06** | 5.04e-06 |
| 7 | rs12188147 | 5 | 26907047 | T | 3226 | 0.51 | 0.38-0.69 | 9.23e-06 | 5.04e-06 |
| 7 | 5:26919594:G:A | 5 | 26919594 | A | 3228 | 0.51 | 0.38-0.69 | 7.76e-06 | 5.55e-06 |
| 7 | rs35759857 | 5 | 26920004 | A | 3228 | 0.51 | 0.38-0.69 | 7.76e-06 | 5.55e-06 |
| **8** | **rs74611811** | **5** | **55568117** | **C** | **3235** | **0.56** | **0.42-0.74** | **4.92e-05** | 2.35e-05 |
| 9 | rs211514 | 5 | 94321669 | G | 3238 | 1.31 | 1.15-1.50 | 4.48e-05 | 1.63e-04 |
| 9 | rs62364694 | 5 | 94336330 | A | 3186 | 1.33 | 1.17-1.52 | 2.14e-05 | 7.37e-05 |
| 9 | rs62364696 | 5 | 94339455 | T | 3248 | 1.31 | 1.15-1.49 | 4.13e-05 | 1.95e-04 |
| 9 | rs309808 | 5 | 94340043 | T | 3235 | 1.3 | 1.14-1.47 | 4.52e-05 | 4.19e-04 |
| 9 | rs309806 | 5 | 94341031 | T | 3234 | 1.29 | 1.14-1.47 | 4.82e-05 | 4.42e-04 |
| 9 | rs35458953 | 5 | 94341126 | T | 3223 | 1.3 | 1.15-1.47 | 3.86e-05 | 4.18e-04 |
| **9** | **rs11957712** | **5** | **94344158** | **T** | **3216** | **1.3** | **1.16-1.46** | **1.03e-05** | 6.71e-05 |
| **10** | **rs201849146** | **6** | **85705310** | **T** | **3170** | **0.56** | **0.43-0.74** | **3.63e-05** | 4.74e-04 |
| **11** | **rs143147350** | **6** | **106900318** | **A** | **3211** | **0.39** | **0.25-0.61** | **4.09e-05** | 5.07e-05 |
| 12 | rs9459086 | 6 | 164818127 | T | 3185 | 0.72 | 0.61-0.84 | 3.75e-05 | 4.05e-04 |
| 12 | rs1856713 | 6 | 164851857 | T | 3214 | 0.71 | 0.61-0.83 | 1.89e-05 | 1.60e-04 |
| 12 | rs5881590 | 6 | 164862072 | C | 3219 | 0.71 | 0.61-0.83 | 1.91e-05 | 1.69e-04 |
| **12** | **6:164876647:T:G** | **6** | **164876647** | **G** | **3247** | **0.71** | **0.61-0.83** | **1.26e-05** | 1.06e-04 |
| 13 | rs28534765 | 7 | 39279013 | G | 3256 | 1.25 | 1.12-1.38 | 3.09e-05 | 1.79e-04 |
| 13 | rs11505524 | 7 | 39280878 | T | 3250 | 0.8 | 0.72-0.89 | 2.02e-05 | 1.30e-04 |
| 13 | rs35439815 | 7 | 39283066 | T | 3251 | 0.8 | 0.72-0.89 | 2.03e-05 | 1.30e-04 |
| **13** | **rs10272803** | **7** | **39285803** | **G** | **3247** | **0.8** | **0.72-0.88** | **1.77e-05** | 1.16e-04 |
| 13 | rs35730750 | 7 | 39287122 | CAAGT | 3241 | 1.28 | 1.15-1.42 | 5.11e-06 | 3.86e-05 |
| 13 | rs7789406 | 7 | 39287983 | A | 3201 | 0.8 | 0.72-0.89 | 2.78e-05 | 1.73e-04 |
| **14** | **rs182081737** | **7** | **83312100** | **A** | **3239** | **0.37** | **0.23-0.60** | **4.84e-05** | 5.09e-05 |
| **15** | **rs139991244** | **7** | **129174247** | **T** | **3158** | **0.65** | **0.53-0.80** | **4.94e-05** | 1.80e-05 |
| **16** | **8:2098794:AT:ATT** | **8** | **2098794** | **ATT** | **3225** | **1.32** | **1.16-1.50** | **2.71e-05** | 2.70e-05 |
| **17** | **8:49309438:C:T** | **8** | **49309438** | **T** | **3233** | **0.41** | **0.27-0.63** | **3.75e-05** | 1.10e-03 |
| **18** | **rs6980703** | **8** | **106126025** | **A** | **3208** | **1.28** | **1.14-1.44** | **3.70e-05** | 3.50e-04 |
| 19 | rs10976477 | 9 | 7652649 | T | 3149 | 0.74 | 0.64-0.85 | 4.60e-05 | 3.31e-05 |
| **19** | **rs10976482** | **9** | **7662414** | **A** | **3216** | **0.72** | **0.63-0.84** | **1.35e-05** | 1.370e-05 |
| 20 | rs7068923 | 10 | 5025483 | G | 3235 | 0.77 | 0.69-0.85 | 7.65e-07 | 9.95e-05 |
| 20 | rs6601887 | 10 | 5025486 | C | 3234 | 0.76 | 0.69-0.85 | 6.19e-07 | 8.52e-05 |
| 20 | rs6601888 | 10 | 5025639 | C | 3235 | 0.77 | 0.69-0.85 | 7.65e-07 | 9.95e-05 |
| 20 | rs7922874 | 10 | 5026017 | T | 3238 | 0.77 | 0.69-0.85 | 8.95e-07 | 1.19e-04 |
| 20 | rs7909151 | 10 | 5026096 | A | 3254 | 0.77 | 0.69-0.86 | 1.18e-06 | 1.44e-04 |
| 20 | rs12250633 | 10 | 5026364 | G | 3237 | 0.77 | 0.69-0.85 | 8.86e-07 | 1.24e-04 |
| 20 | rs11252862 | 10 | 5026823 | C | 3232 | 0.77 | 0.69-0.86 | 1.23e-06 | 1.25e-04 |
| **20** | **rs7914075** | **10** | **5027460** | **G** | **3239** | **0.75** | **0.68-0.84** | **2.41e-07** | 4.03e-05 |
| 20 | rs7915338 | 10 | 5027660 | C | 3255 | 0.76 | 0.68-0.84 | 2.66e-07 | 4.49e-05 |
| 20 | rs7915704 | 10 | 5027931 | C | 3245 | 0.76 | 0.68-0.84 | 3.41e-07 | 5.19e-05 |
| 20 | rs11252864 | 10 | 5027948 | G | 3245 | 0.76 | 0.68-0.84 | 3.41e-07 | 5.19e-05 |
| 20 | rs28851863 | 10 | 5028511 | C | 3239 | 0.75 | 0.68-0.84 | 2.41e-07 | 3.95e-05 |
| 20 | rs71391984 | 10 | 5030618 | AT | 3212 | 0.77 | 0.69-0.85 | 1.09e-06 | 1.32e-04 |
| 20 | rs12355347 | 10 | 5032434 | C | 3150 | 0.77 | 0.69-0.85 | 9.68e-07 | 1.03e-04 |
| 20 | rs11252870 | 10 | 5034852 | A | 3215 | 0.76 | 0.68-0.84 | 4.16e-07 | 6.70e-05 |
| 20 | rs3885232 | 10 | 5035467 | A | 3216 | 0.76 | 0.68-0.85 | 4.71e-07 | 7.33e-05 |
| 20 | rs3927920 | 10 | 5035677 | T | 3214 | 0.76 | 0.68-0.84 | 4.09e-07 | 6.14e-05 |
| 20 | rs4344395 | 10 | 5035678 | A | 3214 | 0.76 | 0.68-0.84 | 4.09e-07 | 6.14e-05 |
| 20 | rs2518039 | 10 | 5036346 | A | 3240 | 0.77 | 0.69-0.85 | 1.14e-06 | 1.290e-04 |
| 20 | rs7073878 | 10 | 5056688 | T | 3106 | 0.79 | 0.71-0.89 | 4.38e-05 | 2.15e-03 |
| **21** | **rs11013175** | **10** | **23177410** | **G** | **3126** | **1.46** | **1.22-1.76** | **4.33e-05** | 4.56e-04 |
| **22** | **rs16926612** | **10** | **71453923** | **A** | **3255** | **1.88** | **1.41-2.52** | **1.89e-05** | 2.24e-05 |
| 22 | rs35729528 | 10 | 71454099 | A | 3254 | 1.88 | 1.40-2.51 | 2.16e-05 | 1.64e-05 |
| 22 | rs34838427 | 10 | 71454295 | A | 3238 | 1.85 | 1.38-2.48 | 3.77e-05 | 1.86e-05 |
| **23** | **11:113587398:ATT:AT** | **11** | **113587398** | **ATT** | **3163** | **0.8** | **0.72-0.89** | **4.61e-05** | 4.61e-05 |
| 24 | rs4385909 | 11 | 124338543 | A | 3254 | 1.24 | 1.12-1.38 | 4.76e-05 | 8.06e-04 |
| 24 | rs4595557 | 11 | 124338664 | C | 3246 | 1.24 | 1.12-1.38 | 4.79e-05 | 9.22e-04 |
| 24 | rs11219669 | 11 | 124341481 | A | 3245 | 1.25 | 1.12-1.39 | 3.59e-05 | 6.54e-04 |
| 24 | rs10736541 | 11 | 124343714 | G | 3256 | 1.24 | 1.12-1.38 | 4.97e-05 | 9.41e-04 |
| 24 | rs1074873 | 11 | 124343736 | T | 3248 | 1.25 | 1.13-1.39 | 2.41e-05 | 6.17e-04 |
| 24 | rs10689492 | 11 | 124362896 | ACT | 3178 | 1.25 | 1.12-1.39 | 4.23e-05 | 6.86e-04 |
| 24 | rs7131070 | 11 | 124370191 | T | 3187 | 0.8 | 0.72-0.89 | 2.72e-05 | 3.68e-05 |
| 24 | rs4282991 | 11 | 124386346 | C | 3234 | 1.25 | 1.13-1.39 | 2.35e-05 | 3.91e-04 |
| **24** | **rs10893236** | **11** | **124386739** | **C** | **3230** | **1.26** | **1.13-1.40** | **2.02e-05** | 4.04e-04 |
| 24 | rs10893237 | 11 | 124386765 | G | 3211 | 1.26 | 1.13-1.40 | 2.33e-05 | 3.74e-04 |
| **25** | **rs1589123** | **12** | **83599960** | **A** | **3244** | **0.75** | **0.66-0.86** | **3.01e-05** | 1.92e-04 |
| **26** | **rs61926457** | **12** | **91346422** | **C** | **3175** | **0.49** | **0.35-0.69** | **3.90e-05** | 1.09e-05 |
| 27 | rs35693771 | 12 | 91449852 | G | 3197 | 0.42 | 0.28-0.63 | 3.24e-05 | 2.53e-05 |
| 27 | 12:91547787:C:A | 12 | 91547787 | A | 3224 | 0.33 | 0.21-0.54 | 6.95e-06 | 6.81e-05 |
| 27 | rs139248283 | 12 | 91618973 | T | 3243 | 0.41 | 0.27-0.62 | 3.79e-05 | 1.22e-03 |
| **27** | **rs140887227** | **12** | **91675946** | **A** | **3237** | **0.39** | **0.25-0.60** | **2.34e-05** | 9.50e-04 |
| 28 | rs941180 | 12 | 124984890 | C | 3253 | 0.66 | 0.54-0.80 | 2.32e-05 | 3.93e-05 |
| **28** | **12:124985150:C:T** | **12** | **124985150** | **T** | **3254** | **0.76** | **0.67-0.86** | **1.95e-05** | 2.48e-04 |
| **29** | **13:56281612:CTTTATTTA:CTTTATTTATTTA** | **13** | **56281612** | **CTTTATTTATTTA** | **3152** | **0.56** | **0.44-0.72** | **4.04e-06** | 1.45e-05 |
| **30** | **rs6492814** | **13** | **96236376** | **G** | **3257** | **1.24** | **1.12-1.38** | **4.34e-05** | 1.01e-03 |
| 30 | rs6492815 | 13 | 96241835 | G | 3227 | 1.24 | 1.12-1.38 | 4.41e-05 | 1.02e-03 |
| **31** | **rs61965297** | **13** | **107091975** | **G** | **3199** | **0.38** | **0.25-0.59** | **1.12e-05** | 7.40e-06 |
| **32** | **rs61986009** | **14** | **59629899** | **A** | **3198** | **0.64** | **0.51-0.79** | **4.90e-05** | 3.52e-05 |
| **33** | **rs8004804** | **14** | **88792303** | **C** | **3192** | **1.28** | **1.14-1.44** | **3.49e-05** | 3.42e-04 |
| **34** | **rs1744269** | **14** | **104546767** | **A** | **3237** | **1.3** | **1.15-1.47** | **1.91e-05** | 5.81e-06 |
| **35** | **rs78027310** | **15** | **95058070** | **C** | **3213** | **2.91** | **1.75-4.83** | **3.63e-05** | 8.42e-04 |
| 35 | rs139905929 | 15 | 95059538 | T | 3213 | 2.91 | 1.75-4.83 | 3.63e-05 | 8.42e-04 |
| **36** | **rs111981919** | **15** | **101086683** | **A** | **3172** | **0.58** | **0.44-0.75** | **4.34e-05** | 3.30e-04 |
| 36 | rs77355140 | 15 | 101088813 | T | 3155 | 0.59 | 0.46-0.76 | 4.64e-05 | 1.46e-04 |
| 36 | rs76517034 | 15 | 101089475 | T | 3154 | 0.59 | 0.46-0.76 | 4.72e-05 | 1.48e-04 |
| 36 | rs4965316 | 15 | 101092226 | C | 3154 | 0.59 | 0.46-0.76 | 4.72e-05 | 1.53e-04 |
| 36 | rs111870044 | 15 | 101092815 | C | 3154 | 0.59 | 0.46-0.76 | 4.72e-05 | 1.53e-04 |
| 36 | rs528265607 | 15 | 101117522 | A | 3153 | 0.54 | 0.42-0.71 | 7.08e-06 | 4.05e-05 |
| 36 | rs145368189 | 15 | 101122426 | T | 3158 | 0.54 | 0.42-0.71 | 6.57e-06 | 3.80e-05 |
| **36** | **rs79835185** | **15** | **101135863** | **A** | **3103** | **0.55** | **0.42-0.72** | **1.76e-05** | 4.69e-05 |
| **37** | **16:27010076:G:A** | **16** | **27010076** | **A** | **3235** | **1.28** | **1.14-1.43** | **3.86e-05** | 1.48e-03 |
| 38 | rs74018931 | 16 | 51755027 | C | 3132 | 1.39 | 1.19-1.62 | 4.03e-05 | 1.82e-03 |
| 38 | rs74018932 | 16 | 51755089 | G | 3132 | 1.39 | 1.19-1.62 | 4.03e-05 | 1.82e-03 |
| 38 | rs56281239 | 16 | 51755747 | T | 3158 | 1.4 | 1.20-1.64 | 2.05e-05 | 1.17e-03 |
| 38 | rs79219580 | 16 | 51756759 | C | 3157 | 1.4 | 1.20-1.64 | 2.51e-05 | 1.36e-03 |
| 38 | rs75182255 | 16 | 51757397 | T | 3145 | 1.39 | 1.18-1.62 | 4.93e-05 | 2.84e-03 |
| 38 | rs59325294 | 16 | 51758339 | C | 3157 | 1.4 | 1.20-1.64 | 2.50e-05 | 1.35e-03 |
| **38** | **rs11407422** | **16** | **51758611** | **AT** | **3121** | **1.45** | **1.23-1.70** | **1.04e-05** | 4.44e-04 |
| 38 | rs150340220 | 16 | 51759205 | TA | 3163 | 1.4 | 1.20-1.64 | 2.36e-05 | 1.32e-03 |
| 38 | rs72213405 | 16 | 51759237 | A | 3158 | 1.4 | 1.20-1.64 | 2.05e-05 | 1.17e-03 |
| 39 | rs77589273 | 18 | 51948350 | G | 3248 | 3.2 | 1.87-5.47 | 2.09e-05 | 2.26e-05 |
| 39 | rs7244796 | 18 | 51965565 | T | 3250 | 3.36 | 1.97-5.73 | 8.91e-06 | 9.92e-06 |
| **39** | **rs111989597** | **18** | **51967716** | **GT** | **3190** | **3.09** | **1.86-5.14** | **1.31e-05** | 1.16e-05 |
| 39 | rs148135376 | 18 | 51980003 | C | 3258 | 2.82 | 1.72-4.62 | 4.14e-05 | 3.97e-05 |
| 39 | rs77423935 | 18 | 51980438 | C | 3258 | 2.82 | 1.72-4.62 | 4.14e-05 | 3.97e-05 |
| 39 | rs77025967 | 18 | 51981553 | T | 3258 | 2.82 | 1.72-4.62 | 4.14e-05 | 3.97e-05 |
| 39 | rs10469029 | 18 | 51985601 | G | 3258 | 2.82 | 1.72-4.62 | 4.14e-05 | 3.97e-05 |
| 39 | rs79967953 | 18 | 51987349 | G | 3258 | 2.82 | 1.72-4.62 | 4.14e-05 | 3.97e-05 |
| 39 | rs77795571 | 18 | 51989575 | G | 3258 | 2.82 | 1.72-4.62 | 4.14e-05 | 3.97e-05 |
| 39 | rs114199484 | 18 | 51992788 | T | 3258 | 2.82 | 1.72-4.62 | 4.14e-05 | 5.30e-05 |
| 39 | rs139266625 | 18 | 51994781 | T | 3258 | 2.82 | 1.72-4.62 | 4.14e-05 | 5.30e-05 |
| 39 | rs76318074 | 18 | 51997175 | T | 3250 | 2.81 | 1.71-4.62 | 4.26e-05 | 5.45e-05 |
| 39 | rs10468878 | 18 | 51998268 | G | 3259 | 2.84 | 1.73-4.67 | 3.48e-05 | 4.52e-05 |
| 39 | rs74696579 | 18 | 51998788 | T | 3259 | 2.84 | 1.73-4.67 | 3.48e-05 | 4.52e-05 |
| 39 | rs149579775 | 18 | 51999478 | AT | 3259 | 2.84 | 1.73-4.67 | 3.48e-05 | 4.52e-05 |
| 39 | rs8090420 | 18 | 52000773 | C | 3259 | 2.84 | 1.73-4.67 | 3.48e-05 | 4.82e-05 |
| 39 | rs8089329 | 18 | 52000774 | T | 3259 | 2.84 | 1.73-4.67 | 3.48e-05 | 4.82e-05 |
| 39 | rs116224434 | 18 | 52002637 | G | 3259 | 2.84 | 1.73-4.67 | 3.48e-05 | 4.82e-05 |
| 39 | rs79815581 | 18 | 52002965 | C | 3259 | 2.84 | 1.73-4.67 | 3.48e-05 | 4.82e-05 |
| 39 | rs115297185 | 18 | 52003248 | G | 3259 | 2.84 | 1.73-4.67 | 3.48e-05 | 4.82e-05 |
| 39 | rs7242585 | 18 | 52007327 | A | 3259 | 2.84 | 1.73-4.67 | 3.48e-05 | 4.82e-05 |
| 39 | rs541543954 | 18 | 52010133 | T | 3259 | 2.84 | 1.73-4.67 | 3.48e-05 | 4.82e-05 |
| 39 | rs147135284 | 18 | 52018638 | G | 3259 | 2.84 | 1.73-4.67 | 3.48e-05 | 4.82e-05 |
| 39 | rs76796794 | 18 | 52019048 | A | 3259 | 2.84 | 1.73-4.67 | 3.48e-05 | 4.82e-05 |
| 39 | 18:52019494:C:T | 18 | 52019494 | T | 3260 | 2.84 | 1.73-4.66 | 3.51e-05 | 4.86e-05 |
| 39 | rs2339997 | 18 | 52020257 | G | 3259 | 2.84 | 1.73-4.67 | 3.48e-05 | 4.82e-05 |
| 39 | rs136381110 | 18 | 52028880 | G | 3253 | 3.06 | 1.82-5.16 | 2.60e-05 | 3.72e-05 |
| 39 | rs77151543 | 18 | 52029749 | T | 3253 | 3.06 | 1.82-5.16 | 2.60e-05 | 3.72e-05 |
| **40** | **rs11665399** | **18** | **65624511** | **T** | **3188** | **1.26** | **1.13-1.40** | **2.23e-05** | 3.19e-04 |
| **41** | **20:4779194:C:G** | **20** | **4779194** | **G** | **3175** | **0.74** | **0.65-0.86** | **4.97e-05** | 1.45e-04 |
| **42** | **rs34185427** | **22** | **17651006** | **AC** | **3207** | **0.6** | **0.47-0.77** | **4.95e-05** | 1.74e-04 |

**Supplementary Table S4: GWAS top SNPs for persistence to methotrexate at three years within the primary analysis cohort.** *Summary of SNPs associated with persistence at one year at a level of p < 5e-5, grouped according to their regional clusters. A regional cluster is defined as all SNPs reaching p < 5e-5 within 200kb of the lead SNP. Bolded SNPs indicate the lead SNP, and p_pool_ denotes p-values in the pooled meta-analysis. Reported positions are per hg19.*

| Region | SNP | Chromosome | Position | Effect Allele | N | OR | 95% CI | P | P_pool_ |
| --- | --- | --- | --- | --- | --- | --- | --- | --- | --- |
| 1 | rs12090529 | 1 | 101707126 | C | 3195 | 1.54 | 1.27-1.88 | 1.71e-05 | 5.95e-05 |
| **1** | **rs3737576** | **1** | **101709563** | **C** | **3213** | **1.55** | **1.27-1.88** | **1.24e-05** | 4.79e-05 |
| 1 | rs138066520 | 1 | 101720606 | C | 3165 | 1.54 | 1.26-1.89 | 2.24e-05 | 8.23e-05 |
| **2** | **rs1539454** | **1** | **219983780** | **C** | **3222** | **0.36** | **0.22-0.59** | **4.45e-05** | 1.20e-03 |
| **3** | **rs4574157** | **2** | **31667880** | **A** | **3221** | **2** | **1.45-2.76** | **2.15e-05** | 1.33e-04 |
| **4** | **rs1452077** | **3** | **62482983** | **C** | **3149** | **1.44** | **1.21-1.71** | **3.87e-05** | 3.81e-04 |
| 5 | rs58755666 | 3 | 124972240 | T | 3194 | 0.78 | 0.70-0.87 | 2.95e-06 | 9.82e-06 |
| 5 | rs4679378 | 3 | 124992550 | T | 3192 | 0.78 | 0.70-0.86 | 1.89e-06 | 6.70e-06 |
| **5** | **rs62270334** | **3** | **125026294** | **T** | **3164** | **0.77** | **0.70-0.86** | **1.56e-06** | 4.58e-06 |
| 5 | rs62270335 | 3 | 125026295 | T | 3164 | 0.77 | 0.70-0.86 | 1.56e-06 | 4.58e-06 |
| 5 | rs62270336 | 3 | 125026298 | T | 3164 | 0.77 | 0.70-0.86 | 1.56e-06 | 4.58e-06 |
| 5 | rs62270346 | 3 | 125027662 | A | 3201 | 0.78 | 0.70-0.87 | 3.37e-06 | 1.11e-05 |
| 5 | rs12485473 | 3 | 125049936 | T | 3210 | 0.78 | 0.71-0.87 | 4.38e-06 | 1.40e-05 |
| 5 | rs7609684 | 3 | 125075013 | A | 3218 | 0.78 | 0.70-0.87 | 2.81e-06 | 9.61e-06 |
| 6 | rs139190198 | 3 | 148588731 | G | 3143 | 2.29 | 1.57-3.35 | 1.82e-05 | 4.03e-05 |
| 6 | rs144007833 | 3 | 148588754 | C | 3143 | 2.29 | 1.57-3.35 | 1.82e-05 | 4.03e-05 |
| 6 | rs148747654 | 3 | 148588757 | C | 3143 | 2.29 | 1.57-3.35 | 1.82e-05 | 4.03e-05 |
| 6 | rs73866681 | 3 | 148591721 | A | 3143 | 2.31 | 1.58-3.38 | 1.45e-05 | 4.92e-05 |
| **6** | **rs77921091** | **3** | **148595453** | **G** | **3156** | **2.32** | **1.59-3.37** | **1.10e-05** | 3.85e-05 |
| **7** | **rs1458819** | **4** | **55954882** | **C** | **3168** | **1.38** | **1.19-1.62** | **4.00e-05** | 3.05e-04 |
| 7 | rs1458831 | 4 | 55958208 | C | 3216 | 1.38 | 1.18-1.61 | 4.11e-05 | 1.89e-04 |
| **8** | **4:60881839:C:G** | **4** | **60881839** | **G** | **3134** | **0.58** | **0.45-0.75** | **4.89e-05** | 2.00e-05 |
| 9 | rs13133181 | 4 | 143610293 | G | 3088 | 1.26 | 1.13-1.40 | 3.02e-05 | 9.71e-05 |
| 9 | rs148442332 | 4 | 143615090 | TTCTC | 3095 | 1.26 | 1.13-1.40 | 3.26e-05 | 9.93e-05 |
| 9 | rs11943397 | 4 | 143617304 | T | 3215 | 1.25 | 1.13-1.38 | 2.47e-05 | 1.01e-04 |
| 9 | rs1899565 | 4 | 143619167 | A | 3192 | 1.24 | 1.12-1.38 | 3.51e-05 | 1.36e-04 |
| 9 | 4:143619586:T:C | 4 | 143619586 | T | 3197 | 1.25 | 1.12-1.38 | 2.91e-05 | 1.29e-04 |
| 9 | rs1961836 | 4 | 143629150 | G | 3214 | 1.3 | 1.17-1.45 | 1.04e-06 | 2.29e-05 |
| 9 | rs12233703 | 4 | 143630177 | A | 3214 | 1.3 | 1.17-1.45 | 1.03e-06 | 2.28e-05 |
| 9 | rs1443187 | 4 | 143631691 | G | 3206 | 1.3 | 1.17-1.45 | 1.73e-06 | 6.48e-05 |
| 9 | rs1443186 | 4 | 143633793 | C | 3190 | 1.3 | 1.17-1.44 | 1.10e-06 | 3.32e-05 |
| 9 | rs1443185 | 4 | 143633841 | T | 3187 | 1.3 | 1.17-1.44 | 1.04e-06 | 3.17e-05 |
| 9 | rs12644329 | 4 | 143634746 | G | 3075 | 1.29 | 1.16-1.44 | 2.22e-06 | 6.24e-05 |
| 9 | rs1373034 | 4 | 143635257 | C | 3210 | 1.28 | 1.14-1.42 | 1.40e-05 | 5.92e-04 |
| 9 | rs11100754 | 4 | 143637428 | T | 3084 | 1.3 | 1.17-1.44 | 1.38e-06 | 4.25e-05 |
| 9 | rs6813458 | 4 | 143638882 | G | 3197 | 1.3 | 1.17-1.44 | 9.25e-07 | 2.87e-05 |
| 9 | rs4690698 | 4 | 143639211 | T | 3189 | 1.28 | 1.15-1.43 | 8.59e-06 | 2.06e-04 |
| 9 | rs4690699 | 4 | 143639498 | C | 3191 | 1.3 | 1.17-1.45 | 8.28e-07 | 2.63e-05 |
| 9 | rs1373032 | 4 | 143640523 | C | 3191 | 1.3 | 1.17-1.45 | 8.28e-07 | 2.63e-05 |
| 9 | rs7666140 | 4 | 143641202 | T | 3205 | 1.28 | 1.14-1.43 | 1.25e-05 | 5.46e-04 |
| 9 | rs7376475 | 4 | 143641788 | T | 3196 | 1.29 | 1.16-1.44 | 1.90e-06 | 9.34e-05 |
| **9** | **rs13141925** | **4** | **143642099** | **T** | **3191** | **1.3** | **1.17-1.45** | **8.24e-07** | 2.62e-05 |
| 9 | rs4690725 | 4 | 143643387 | G | 3205 | 1.28 | 1.14-1.43 | 1.25e-05 | 5.45e-04 |
| 9 | rs4690700 | 4 | 143643588 | C | 3205 | 1.28 | 1.14-1.43 | 1.25e-05 | 5.45e-04 |
| 9 | rs1822362 | 4 | 143644115 | C | 3205 | 1.28 | 1.14-1.43 | 1.25e-05 | 5.45e-04 |
| 9 | rs1822361 | 4 | 143644163 | A | 3191 | 1.3 | 1.17-1.44 | 1.17e-06 | 6.43e-05 |
| 9 | rs5862608 | 4 | 143644186 | A | 3202 | 1.28 | 1.15-1.43 | 1.19e-05 | 5.00e-04 |
| 9 | rs10857399 | 4 | 143644561 | A | 3215 | 1.29 | 1.16-1.43 | 2.49e-06 | 1.14e-04 |
| 9 | rs10024696 | 4 | 143647878 | T | 3215 | 1.29 | 1.16-1.43 | 2.49e-06 | 1.14e-04 |
| 9 | rs4690726 | 4 | 143648219 | A | 3215 | 1.29 | 1.16-1.43 | 2.49e-06 | 1.14e-04 |
| 9 | 4:143648579:C:G | 4 | 143648579 | C | 3176 | 1.29 | 1.15-1.44 | 8.45e-06 | 4.95e-04 |
| 9 | rs59830705 | 4 | 143648644 | G | 3203 | 1.27 | 1.14-1.42 | 2.38e-05 | 8.86e-04 |
| 9 | rs1373031 | 4 | 143649411 | G | 3215 | 1.29 | 1.16-1.43 | 2.49e-06 | 1.14e-04 |
| 9 | rs1838082 | 4 | 143649618 | C | 3211 | 1.29 | 1.16-1.43 | 2.85e-06 | 1.27e-04 |
| 9 | rs5862609 | 4 | 143650102 | TA | 3216 | 1.27 | 1.14-1.42 | 1.63e-05 | 6.59e-04 |
| 9 | rs1867214 | 4 | 143650258 | T | 3215 | 1.29 | 1.16-1.43 | 2.49e-06 | 1.14e-04 |
| 9 | rs13117185 | 4 | 143650322 | C | 3215 | 1.29 | 1.16-1.43 | 2.49e-06 | 1.14e-04 |
| 9 | rs111268660 | 4 | 143650666 | G | 3198 | 1.28 | 1.15-1.42 | 3.75e-06 | 1.64e-04 |
| 9 | rs12640389 | 4 | 143652106 | C | 3215 | 1.29 | 1.16-1.43 | 2.49e-06 | 1.10e-04 |
| 9 | rs540166018 | 4 | 143652118 | T | 3215 | 1.29 | 1.16-1.43 | 2.49e-06 | 1.10e-04 |
| 9 | rs1965828 | 4 | 143652317 | T | 3215 | 1.29 | 1.16-1.43 | 2.49e-06 | 1.10e-04 |
| 9 | rs1465962 | 4 | 143652569 | A | 3213 | 1.28 | 1.16-1.43 | 3.04e-06 | 1.23e-04 |
| 9 | rs1373030 | 4 | 143652701 | T | 3216 | 1.28 | 1.14-1.43 | 1.24e-05 | 5.36e-04 |
| 9 | rs35531661 | 4 | 143652924 | A | 3114 | 1.27 | 1.14-1.42 | 1.91e-05 | 6.44e-04 |
| 9 | 4:143652993:C:T | 4 | 143652993 | T | 3216 | 1.29 | 1.16-1.43 | 2.06e-06 | 9.44e-05 |
| 9 | rs2165819 | 4 | 143654534 | G | 3219 | 1.28 | 1.15-1.43 | 7.01e-06 | 1.92e-04 |
| 9 | rs331939 | 4 | 143654889 | A | 3217 | 1.29 | 1.16-1.43 | 1.87e-06 | 8.76e-05 |
| 9 | 4:143655898:G:A | 4 | 143655898 | A | 3213 | 1.28 | 1.16-1.43 | 3.19e-06 | 1.33e-04 |
| 9 | rs179508 | 4 | 143657575 | C | 3180 | 1.3 | 1.17-1.44 | 1.30e-06 | 3.43e-05 |
| 9 | rs331942 | 4 | 143657595 | T | 3222 | 1.29 | 1.16-1.43 | 2.12e-06 | 8.99e-05 |
| 9 | rs331943 | 4 | 143658496 | G | 3221 | 1.28 | 1.14-1.43 | 1.30e-05 | 5.55e-04 |
| 9 | rs167960 | 4 | 143659234 | G | 3219 | 1.28 | 1.14-1.43 | 1.32e-05 | 5.30e-04 |
| 9 | rs3113593 | 4 | 143659668 | T | 3221 | 1.29 | 1.16-1.43 | 3.13e-06 | 1.02e-04 |
| 9 | rs2589994 | 4 | 143660160 | A | 3220 | 1.29 | 1.16-1.43 | 2.08e-06 | 8.90e-05 |
| 9 | rs1443183 | 4 | 143660758 | A | 3218 | 1.29 | 1.16-1.43 | 1.90e-06 | 8.29e-05 |
| 9 | rs331949 | 4 | 143663206 | C | 3217 | 1.3 | 1.17-1.44 | 1.04e-06 | 2.79e-05 |
| 9 | rs331950 | 4 | 143663539 | A | 3161 | 1.3 | 1.17-1.44 | 1.28e-06 | 3.41e-05 |
| 9 | rs331951 | 4 | 143664834 | C | 3217 | 1.29 | 1.16-1.43 | 3.07e-06 | 1.01e-04 |
| 9 | rs331952 | 4 | 143665993 | T | 3217 | 1.28 | 1.14-1.43 | 1.28e-05 | 5.52e-04 |
| 9 | rs11378725 | 4 | 143666158 | GT | 3218 | 1.28 | 1.15-1.42 | 8.50e-06 | 4.64e-04 |
| 9 | rs331953 | 4 | 143667098 | C | 3218 | 1.28 | 1.15-1.42 | 8.50e-06 | 4.64e-04 |
| 9 | rs331954 | 4 | 143667593 | G | 3217 | 1.28 | 1.14-1.43 | 1.28e-05 | 5.52e-04 |
| 9 | rs331955 | 4 | 143667954 | G | 3217 | 1.3 | 1.17-1.44 | 1.04e-06 | 2.79e-05 |
| 9 | rs201955412 | 4 | 143668329 | GA | 3217 | 1.3 | 1.17-1.44 | 1.04e-06 | 2.79e-05 |
| 9 | rs331956 | 4 | 143668365 | A | 3217 | 1.28 | 1.14-1.43 | 1.28e-05 | 5.50e-04 |
| 9 | rs331957 | 4 | 143668523 | A | 3182 | 1.29 | 1.17-1.44 | 1.38e-06 | 3.59e-05 |
| 9 | rs111391726 | 4 | 143669028 | TG | 3215 | 1.29 | 1.16-1.44 | 2.68e-06 | 9.10e-05 |
| 9 | rs331958 | 4 | 143669498 | A | 3217 | 1.28 | 1.14-1.43 | 1.28e-05 | 5.51e-04 |
| 9 | rs331959 | 4 | 143669935 | C | 3217 | 1.28 | 1.14-1.43 | 1.29e-05 | 5.54e-04 |
| 9 | rs331960 | 4 | 143670256 | T | 3217 | 1.28 | 1.14-1.43 | 1.28e-05 | 5.51e-04 |
| 9 | rs10637586 | 4 | 143671024 | TCAAA | 3217 | 1.3 | 1.17-1.44 | 1.04e-06 | 2.79e-05 |
| 9 | rs331961 | 4 | 143671111 | G | 3217 | 1.28 | 1.14-1.43 | 1.28e-05 | 5.51e-04 |
| 9 | rs35609076 | 4 | 143672642 | ACT | 3217 | 1.29 | 1.16-1.43 | 1.84e-06 | 8.08e-05 |
| 9 | rs331962 | 4 | 143673174 | C | 3217 | 1.28 | 1.14-1.43 | 1.28e-05 | 5.51e-04 |
| 9 | rs331963 | 4 | 143673851 | A | 3216 | 1.3 | 1.17-1.44 | 1.03e-06 | 2.76e-05 |
| 9 | rs331964 | 4 | 143674396 | C | 3217 | 1.28 | 1.14-1.43 | 1.29e-05 | 5.52e-04 |
| 9 | rs331965 | 4 | 143674647 | G | 3217 | 1.28 | 1.14-1.43 | 1.29e-05 | 5.52e-04 |
| 9 | rs331966 | 4 | 143675717 | C | 3169 | 1.27 | 1.15-1.42 | 5.23e-06 | 1.21e-04 |
| 9 | rs331948 | 4 | 143679854 | C | 3203 | 1.28 | 1.16-1.42 | 3.13e-06 | 6.83e-05 |
| 9 | rs331947 | 4 | 143680185 | A | 3205 | 1.26 | 1.13-1.41 | 3.67e-05 | 1.22e-03 |
| 9 | rs331946 | 4 | 143680948 | A | 3205 | 1.26 | 1.13-1.41 | 3.67e-05 | 1.22e-03 |
| 9 | rs331945 | 4 | 143683033 | G | 3204 | 1.26 | 1.13-1.41 | 3.58e-05 | 1.12e-03 |
| 9 | rs191762 | 4 | 143683702 | C | 3179 | 1.27 | 1.15-1.41 | 7.08e-06 | 1.29e-04 |
| 9 | rs331944 | 4 | 143686977 | T | 3205 | 1.26 | 1.13-1.41 | 3.66e-05 | 1.22e-03 |
| 9 | rs17016768 | 4 | 143693739 | T | 3204 | 1.26 | 1.13-1.41 | 3.58e-05 | 1.20e-03 |
| 9 | rs72347187 | 4 | 143695633 | T | 3205 | 1.26 | 1.13-1.41 | 3.66e-05 | 1.22e-03 |
| 9 | rs72728641 | 4 | 143696600 | A | 3205 | 1.26 | 1.13-1.41 | 3.66e-05 | 1.22e-03 |
| 9 | rs141475479 | 4 | 143696638 | GTTCCC | 3176 | 1.28 | 1.15-1.42 | 7.19e-06 | 1.06e-04 |
| 9 | rs56332157 | 4 | 143696835 | C | 3205 | 1.26 | 1.13-1.41 | 3.68e-05 | 1.22e-03 |
| 9 | rs56011189 | 4 | 143696908 | T | 3205 | 1.26 | 1.13-1.41 | 3.68e-05 | 1.22e-03 |
| 9 | rs6537124 | 4 | 143699841 | G | 3171 | 1.28 | 1.15-1.42 | 4.78e-06 | 9.79e-05 |
| 9 | rs6852570 | 4 | 143700358 | T | 3171 | 1.28 | 1.15-1.42 | 4.78e-06 | 9.79e-05 |
| 9 | rs34442253 | 4 | 143700819 | T | 3191 | 1.28 | 1.16-1.43 | 3.04e-06 | 6.73e-05 |
| 9 | rs11726381 | 4 | 143701688 | G | 3195 | 1.26 | 1.13-1.41 | 4.45e-05 | 1.42e-03 |
| 9 | rs17732408 | 4 | 143702257 | C | 3195 | 1.26 | 1.13-1.41 | 4.45e-05 | 1.42e-03 |
| 9 | rs6816424 | 4 | 143705445 | G | 3191 | 1.28 | 1.16-1.43 | 3.04e-06 | 6.74e-05 |
| 9 | rs72728649 | 4 | 143707536 | G | 3181 | 1.26 | 1.13-1.41 | 4.56e-05 | 1.55e-03 |
| 9 | rs966457 | 4 | 143708318 | C | 3188 | 1.29 | 1.16-1.43 | 2.42e-06 | 6.55e-05 |
| 9 | rs72728651 | 4 | 143711915 | T | 3197 | 1.26 | 1.13-1.41 | 3.24e-05 | 1.07e-03 |
| 9 | rs10676551 | 4 | 143716110 | CAT | 3141 | 1.28 | 1.15-1.42 | 6.39e-06 | 8.42e-05 |
| 9 | rs6831927 | 4 | 143717164 | T | 3143 | 1.29 | 1.16-1.43 | 2.97e-06 | 3.81e-05 |
| 9 | rs72728654 | 4 | 143717568 | A | 3198 | 1.27 | 1.13-1.41 | 2.76e-05 | 8.48e-04 |
| 9 | rs6819498 | 4 | 143717643 | C | 3134 | 1.28 | 1.15-1.42 | 4.40e-06 | 6.48e-05 |
| 9 | rs199741935 | 4 | 143718020 | G | 3198 | 1.27 | 1.14-1.42 | 1.54e-05 | 6.98e-04 |
| 9 | rs72728658 | 4 | 143718925 | A | 3198 | 1.27 | 1.14-1.42 | 1.55e-05 | 7.02e-04 |
| 9 | rs6845583 | 4 | 143719082 | T | 3153 | 1.29 | 1.16-1.43 | 3.03e-06 | 4.71e-05 |
| 9 | rs6844306 | 4 | 143719195 | C | 3100 | 1.29 | 1.16-1.43 | 3.38e-06 | 5.32e-05 |
| 9 | rs17016777 | 4 | 143721036 | A | 3195 | 1.29 | 1.16-1.43 | 3.05e-06 | 1.14e-04 |
| 9 | rs17016779 | 4 | 143722081 | G | 3195 | 1.27 | 1.14-1.42 | 1.17e-05 | 5.94e-04 |
| 9 | rs28420913 | 4 | 143722511 | A | 3153 | 1.29 | 1.16-1.43 | 1.86e-06 | 3.28e-05 |
| 9 | rs72728667 | 4 | 143722905 | T | 3195 | 1.27 | 1.14-1.42 | 1.17e-05 | 5.94e-04 |
| 9 | rs13435565 | 4 | 143722951 | C | 3091 | 1.28 | 1.16-1.43 | 3.59e-06 | 5.82e-05 |
| 9 | rs12509304 | 4 | 143723264 | G | 3195 | 1.27 | 1.14-1.42 | 1.17e-05 | 5.93e-04 |
| 9 | rs7667183 | 4 | 143723571 | C | 3198 | 1.29 | 1.16-1.43 | 2.93e-06 | 1.14e-04 |
| 9 | rs76149047 | 4 | 143724639 | AT | 3200 | 1.28 | 1.14-1.42 | 1.57e-05 | 5.76e-04 |
| 9 | rs75832326 | 4 | 143724815 | A | 3136 | 1.29 | 1.15-1.44 | 6.86e-06 | 2.65e-04 |
| 9 | rs112854762 | 4 | 143725173 | T | 3199 | 1.28 | 1.14-1.42 | 1.50e-05 | 5.55e-04 |
| 9 | rs11723982 | 4 | 143726017 | T | 3195 | 1.27 | 1.14-1.42 | 1.17e-05 | 5.91e-04 |
| 9 | rs11724017 | 4 | 143726072 | A | 3200 | 1.28 | 1.14-1.42 | 1.57e-05 | 5.76e-04 |
| 9 | rs17016789 | 4 | 143726158 | G | 3202 | 1.27 | 1.14-1.42 | 1.81e-05 | 6.41e-04 |
| 9 | rs7688275 | 4 | 143727241 | G | 3198 | 1.29 | 1.16-1.43 | 2.93e-06 | 1.15e-04 |
| 9 | rs57179728 | 4 | 143728949 | TAA | 3176 | 1.28 | 1.15-1.42 | 4.03e-06 | 1.49e-04 |
| 9 | rs72175284 | 4 | 143729485 | A | 3195 | 1.27 | 1.14-1.42 | 1.17e-05 | 5.92e-04 |
| 9 | rs7679151 | 4 | 143730983 | C | 3202 | 1.27 | 1.14-1.42 | 1.81e-05 | 6.40e-04 |
| 9 | rs7695461 | 4 | 143731041 | T | 3199 | 1.28 | 1.14-1.42 | 1.50e-05 | 5.53e-04 |
| 9 | rs11933763 | 4 | 143732426 | A | 3181 | 1.28 | 1.15-1.42 | 4.95e-06 | 1.66e-04 |
| 9 | rs34831472 | 4 | 143733303 | A | 3202 | 1.27 | 1.14-1.42 | 1.81e-05 | 6.04e-04 |
| 9 | rs72728681 | 4 | 143733469 | T | 3200 | 1.28 | 1.14-1.42 | 1.56e-05 | 5.39e-04 |
| 9 | rs7668552 | 4 | 143733966 | A | 3203 | 1.27 | 1.14-1.42 | 1.88e-05 | 6.22e-04 |
| 9 | rs7668786 | 4 | 143734092 | C | 3203 | 1.27 | 1.14-1.42 | 1.88e-05 | 6.22e-04 |
| 9 | rs17732791 | 4 | 143735198 | T | 3208 | 1.28 | 1.15-1.43 | 5.39e-06 | 1.31e-04 |
| 9 | rs10519666 | 4 | 143735270 | A | 3206 | 1.27 | 1.14-1.42 | 1.78e-05 | 5.95e-04 |
| 9 | rs72728686 | 4 | 143735637 | A | 3203 | 1.27 | 1.14-1.42 | 1.88e-05 | 6.22e-04 |
| 9 | rs1992417 | 4 | 143736804 | A | 3221 | 1.27 | 1.14-1.42 | 8.96e-06 | 1.94e-04 |
| 9 | rs1992418 | 4 | 143737159 | A | 3222 | 1.28 | 1.15-1.42 | 7.70e-06 | 1.83e-04 |
| 9 | rs113347350 | 4 | 143737955 | CTATT | 3167 | 1.27 | 1.15-1.42 | 9.35e-06 | 1.87e-04 |
| 9 | rs6838663 | 4 | 143740940 | T | 3163 | 1.26 | 1.13-1.41 | 2.59e-05 | 8.35e-04 |
| 9 | 4:143742567:A:C | 4 | 143742567 | C | 3098 | 1.28 | 1.15-1.42 | 5.69e-06 | 1.82e-04 |
| 9 | rs7660533 | 4 | 143744809 | A | 3135 | 1.27 | 1.14-1.41 | 1.08e-05 | 2.92e-04 |
| 9 | rs6823627 | 4 | 143745608 | A | 3196 | 1.26 | 1.13-1.40 | 3.40e-05 | 1.08e-03 |
| 9 | rs1444949 | 4 | 143747354 | C | 3220 | 1.25 | 1.12-1.39 | 4.90e-05 | 1.45e-03 |
| 9 | rs571053259 | 4 | 143752003 | C | 3151 | 1.27 | 1.14-1.41 | 1.04e-05 | 2.45e-04 |
| 9 | rs1441418 | 4 | 143756832 | T | 3139 | 1.27 | 1.14-1.41 | 8.55e-06 | 1.95e-04 |
| 9 | rs6811022 | 4 | 143759353 | C | 3152 | 1.25 | 1.12-1.38 | 3.33e-05 | 6.22e-04 |
| 9 | rs1444950 | 4 | 143761816 | A | 3154 | 1.27 | 1.14-1.41 | 9.88e-06 | 2.17e-04 |
| 9 | rs7685997 | 4 | 143762382 | G | 3189 | 1.26 | 1.13-1.40 | 3.40e-05 | 7.94e-04 |
| 9 | rs72728700 | 4 | 143762547 | A | 3189 | 1.26 | 1.13-1.40 | 3.40e-05 | 7.94e-04 |
| 9 | rs13122287 | 4 | 143762778 | C | 3154 | 1.27 | 1.14-1.41 | 9.88e-06 | 2.17e-04 |
| 9 | rs1992652 | 4 | 143763356 | C | 3189 | 1.26 | 1.13-1.40 | 3.40e-05 | 7.94e-04 |
| 9 | rs35407959 | 4 | 143763682 | TA | 3153 | 1.27 | 1.14-1.41 | 9.57e-06 | 2.16e-04 |
| 9 | rs111379736 | 4 | 143763739 | A | 3189 | 1.26 | 1.13-1.40 | 3.33e-05 | 7.83e-04 |
| 9 | rs6831204 | 4 | 143764513 | A | 3161 | 1.25 | 1.13-1.39 | 2.07e-05 | 3.67e-04 |
| 9 | rs11444297 | 4 | 143764851 | CT | 3170 | 1.25 | 1.13-1.39 | 2.24e-05 | 3.89e-04 |
| 9 | rs58445560 | 4 | 143765580 | G | 3187 | 1.26 | 1.13-1.40 | 3.88e-05 | 9.29e-04 |
| 9 | rs9994364 | 4 | 143765773 | A | 3152 | 1.27 | 1.14-1.41 | 9.08e-06 | 2.11e-04 |
| 9 | rs60263370 | 4 | 143766164 | G | 3187 | 1.26 | 1.13-1.40 | 3.88e-05 | 9.29e-04 |
| 9 | rs6846609 | 4 | 143767288 | T | 3186 | 1.25 | 1.13-1.40 | 4.05e-05 | 9.61e-04 |
| **10** | **rs28924068** | **4** | **144381463** | **G** | **3102** | **1.5** | **1.24-1.81** | **2.80e-05** | 8.65e-04 |
| **11** | **rs142917406** | **4** | **190166555** | **C** | **3139** | **1.93** | **1.41-2.63** | **3.45e-05** | 8.50e-04 |
| **12** | **rs2962554** | **5** | **53889651** | **C** | **3163** | **0.81** | **0.73-0.90** | **4.33e-05** | 8.75e-05 |
| 12 | rs2962555 | 5 | 53891524 | A | 3163 | 0.81 | 0.73-0.90 | 4.33e-05 | 8.75e-05 |
| 12 | rs2548625 | 5 | 53891608 | A | 3163 | 0.81 | 0.73-0.90 | 4.33e-05 | 8.75e-05 |
| **13** | **rs3925114** | **5** | **62034844** | **T** | **3213** | **1.24** | **1.12-1.38** | **2.34e-05** | 7.18e-05 |
| **14** | **rs201250** | **6** | **10013636** | **C** | **3222** | **0.78** | **0.69-0.88** | **3.83e-05** | 5.10e-05 |
| **15** | **rs35664708** | **6** | **135539103** | **A** | **3214** | **0.54** | **0.41-0.72** | **1.48e-05** | 1.07e-05 |
| 16 | rs17080185 | 6 | 149256668 | A | 3200 | 0.45 | 0.32-0.65 | 1.49e-05 | 1.25e-05 |
| 16 | rs17080191 | 6 | 149256872 | A | 3207 | 0.42 | 0.29-0.61 | 4.15e-06 | 3.70e-06 |
| 16 | rs111969093 | 6 | 149257893 | A | 3214 | 0.44 | 0.31-0.63 | 7.26e-06 | 4.24e-06 |
| 16 | rs200993430 | 6 | 149264277 | A | 3218 | 0.44 | 0.31-0.63 | 6.64e-06 | 3.25e-06 |
| 16 | rs77744262 | 6 | 149266996 | A | 3221 | 0.44 | 0.31-0.63 | 5.79e-06 | 3.41e-06 |
| 16 | 6:149268845:C:T | 6 | 149268845 | T | 3222 | 0.44 | 0.31-0.63 | 5.68e-06 | 3.35e-06 |
| 16 | rs17080393 | 6 | 149268991 | G | 3222 | 0.44 | 0.31-0.63 | 5.68e-06 | 3.35e-06 |
| 16 | rs112978375 | 6 | 149270356 | C | 3222 | 0.44 | 0.31-0.63 | 5.68e-06 | 3.35e-06 |
| **16** | **rs77668163** | **6** | **149271334** | **T** | **3216** | **0.41** | **0.29-0.60** | **2.42e-06** | 1.50e-06 |
| 16 | rs112382059 | 6 | 149271462 | A | 3216 | 0.41 | 0.29-0.60 | 2.42e-06 | 1.50e-06 |
| **17** | **rs113502303** | **6** | **158262608** | **T** | **3106** | **1.89** | **1.44-2.46** | **3.57e-06** | 1.48e-05 |
| 17 | rs77052819 | 6 | 158281259 | T | 3110 | 1.89 | 1.47-2.44 | 6.16e-07 | 3.57e-06 |
| **18** | **rs59933387** | **6** | **166082502** | **T** | **3218** | **2.16** | **1.49-3.12** | **4.30e-05** | 2.53e-05 |
| **19** | **rs2023593** | **6** | **167625287** | **T** | **3165** | **0.52** | **0.38-0.70** | **2.49e-05** | 3.58e-05 |
| **20** | **7:50473082:C:T** | **7** | **50473082** | **T** | **3221** | **1.78** | **1.36-2.34** | **3.03e-05** | 3.19e-05 |
| **21** | **rs79563067** | **7** | **81722990** | **A** | **3132** | **1.43** | **1.21-1.70** | **4.60e-05** | 2.35e-05 |
| 22 | rs2581581 | 8 | 2124471 | C | 3187 | 0.78 | 0.69-0.87 | 2.60e-05 | 6.02e-05 |
| 22 | rs67020903 | 8 | 2155392 | T | 3149 | 0.78 | 0.69-0.88 | 4.07e-05 | 7.15e-05 |
| **22** | **rs10503173** | **8** | **2162306** | **A** | **3207** | **0.77** | **0.69-0.87** | **2.47e-05** | 4.94e-05 |
| 22 | 8:2164005:G:A | 8 | 2164005 | A | 3213 | 0.77 | 0.69-0.87 | 2.76e-05 | 4.23e-05 |
| **23** | **rs144251294** | **9** | **20319500** | **A** | **3163** | **0.31** | **0.18-0.55** | **4.45e-05** | 4.45e-05 |
| **24** | **rs7035774** | **9** | **107270669** | **T** | **3135** | **0.8** | **0.72-0.89** | **1.48e-05** | 4.53e-05 |
| 24 | rs10761028 | 9 | 107274204 | G | 3137 | 0.8 | 0.72-0.89 | 1.56e-05 | 5.00e-05 |
| 25 | rs78022238 | 9 | 116043654 | G | 3221 | 1.5 | 1.23-1.82 | 4.72e-05 | 4.46e-04 |
| 25 | rs74627363 | 9 | 116051226 | C | 3218 | 1.5 | 1.23-1.82 | 4.78e-05 | 4.51e-04 |
| 25 | rs10981718 | 9 | 116052451 | C | 3216 | 1.51 | 1.24-1.83 | 3.26e-05 | 3.31e-04 |
| 25 | 9:116052538:G:A | 9 | 116052538 | A | 3216 | 1.51 | 1.24-1.83 | 3.26e-05 | 3.31e-04 |
| 25 | rs2418249 | 9 | 116054040 | T | 3204 | 1.51 | 1.24-1.83 | 3.18e-05 | 3.24e-04 |
| 25 | rs117673249 | 9 | 116054246 | G | 3204 | 1.51 | 1.24-1.83 | 3.18e-05 | 3.24e-04 |
| 25 | rs10981721 | 9 | 116057144 | G | 3218 | 1.51 | 1.24-1.83 | 3.60e-05 | 3.58e-04 |
| 25 | rs59841233 | 9 | 116061671 | T | 3220 | 1.5 | 1.24-1.83 | 3.81e-05 | 3.75e-04 |
| 25 | 9:116062597:T:C | 9 | 116062597 | C | 3218 | 1.51 | 1.24-1.83 | 3.60e-05 | 3.58e-04 |
| 25 | rs10981725 | 9 | 116063746 | G | 3217 | 1.5 | 1.24-1.83 | 3.97e-05 | 3.88e-04 |
| 25 | rs71503561 | 9 | 116069947 | A | 3220 | 1.5 | 1.24-1.82 | 3.89e-05 | 3.64e-04 |
| 25 | rs10817477 | 9 | 116075797 | C | 3216 | 1.53 | 1.25-1.87 | 3.45e-05 | 3.99e-04 |
| 25 | rs34294772 | 9 | 116077238 | T | 3222 | 1.54 | 1.26-1.89 | 2.61e-05 | 3.06e-04 |
| 25 | rs12684385 | 9 | 116077694 | G | 3222 | 1.54 | 1.26-1.89 | 2.61e-05 | 3.06e-04 |
| 25 | rs2296076 | 9 | 116079271 | C | 3222 | 1.54 | 1.26-1.89 | 2.61e-05 | 3.06e-04 |
| 25 | rs2296075 | 9 | 116079431 | T | 3213 | 1.54 | 1.26-1.89 | 2.51e-05 | 2.98e-04 |
| **25** | **rs3810920** | **9** | **116091077** | **G** | **3206** | **1.54** | **1.26-1.89** | **2.19e-05** | 3.02e-04 |
| 25 | rs118173698 | 9 | 116092365 | C | 3206 | 1.52 | 1.24-1.87 | 4.90e-05 | 5.13e-04 |
| 25 | rs16932730 | 9 | 116093503 | A | 3208 | 1.55 | 1.27-1.90 | 2.31e-05 | 2.79e-04 |
| 25 | rs35772163 | 9 | 116093837 | G | 3209 | 1.54 | 1.26-1.89 | 2.76e-05 | 3.22e-04 |
| 25 | rs10981734 | 9 | 116094706 | T | 3209 | 1.54 | 1.26-1.89 | 2.76e-05 | 3.22e-04 |
| 25 | rs10981736 | 9 | 116098701 | C | 3207 | 1.53 | 1.25-1.88 | 4.03e-05 | 4.38e-04 |
| 25 | rs16932806 | 9 | 116100368 | C | 3207 | 1.53 | 1.25-1.88 | 4.03e-05 | 4.38e-04 |
| 25 | rs34429100 | 9 | 116104933 | A | 3205 | 1.54 | 1.26-1.89 | 3.43e-05 | 4.60e-04 |
| 25 | rs35275471 | 9 | 116105031 | G | 3205 | 1.54 | 1.26-1.89 | 3.42e-05 | 4.60e-04 |
| 25 | rs13289574 | 9 | 116106759 | A | 3205 | 1.54 | 1.26-1.89 | 3.43e-05 | 4.60e-04 |
| 25 | rs10981754 | 9 | 116107380 | T | 3205 | 1.54 | 1.26-1.89 | 3.42e-05 | 4.60e-04 |
| 25 | rs7850003 | 9 | 116108231 | T | 3212 | 1.54 | 1.26-1.88 | 3.07e-05 | 4.20e-04 |
| 25 | rs35860025 | 9 | 116108768 | G | 3208 | 1.52 | 1.24-1.87 | 4.99e-05 | 5.21e-04 |
| 25 | rs28480972 | 9 | 116109597 | T | 3209 | 1.52 | 1.24-1.87 | 4.91e-05 | 5.14e-04 |
| 25 | rs34289746 | 9 | 116109669 | C | 3209 | 1.52 | 1.24-1.87 | 4.91e-05 | 5.14e-04 |
| 25 | rs61161242 | 9 | 116109701 | A | 3209 | 1.52 | 1.24-1.87 | 4.91e-05 | 5.14e-04 |
| 25 | rs34749539 | 9 | 116110744 | G | 3208 | 1.52 | 1.24-1.87 | 4.99e-05 | 5.20e-04 |
| 26 | rs76538611 | 11 | 5148336 | T | 3222 | 0.44 | 0.29-0.65 | 4.91e-05 | 1.35e-05 |
| **26** | **rs78577958** | **11** | **5148895** | **C** | **3211** | **0.44** | **0.29-0.65** | **4.85e-05** | 2.11e-05 |
| **27** | **rs36027425** | **11** | **18809488** | **A** | **3060** | **0.78** | **0.69-0.88** | **4.85e-05** | 4.64e-06 |
| 28 | rs2305169 | 11 | 36298101 | C | 3199 | 0.8 | 0.73-0.89 | 2.73e-05 | 1.92e-03 |
| **28** | **11:36311014:G:T** | **11** | **36311014** | **T** | **3222** | **0.8** | **0.72-0.89** | **1.57e-05** | 1.20e-03 |
| 28 | rs9888213 | 11 | 36314646 | G | 3147 | 0.8 | 0.72-0.89 | 2.22e-05 | 1.36e-03 |
| 28 | rs7104737 | 11 | 36315189 | A | 3169 | 0.79 | 0.71-0.88 | 6.20e-06 | 6.10e-04 |
| 28 | rs12294763 | 11 | 36325764 | A | 3194 | 1.26 | 1.13-1.40 | 3.13e-05 | 3.21e-03 |
| **29** | **rs75091876** | **11** | **69584735** | **T** | **3220** | **1.81** | **1.37-2.39** | **2.87e-05** | 1.07e-04 |
| 30 | rs9633921 | 11 | 124325984 | A | 3213 | 1.25 | 1.13-1.38 | 2.15e-05 | 1.86e-04 |
| 30 | rs4608094 | 11 | 124327131 | G | 3206 | 1.24 | 1.12-1.38 | 2.65e-05 | 2.22e-04 |
| 30 | rs12294473 | 11 | 124329612 | G | 3209 | 1.24 | 1.12-1.38 | 2.53e-05 | 2.13e-04 |
| 30 | rs12281391 | 11 | 124329615 | T | 3209 | 1.24 | 1.12-1.38 | 2.53e-05 | 2.13e-04 |
| 30 | rs5795420 | 11 | 124337865 | GCT | 3212 | 1.24 | 1.12-1.38 | 2.40e-05 | 2.62e-04 |
| 30 | rs4360714 | 11 | 124337885 | T | 3212 | 1.24 | 1.12-1.38 | 2.40e-05 | 2.62e-04 |
| 30 | 11:124338177:G:C | 11 | 124338177 | C | 3212 | 1.24 | 1.12-1.38 | 2.40e-05 | 2.62e-04 |
| 30 | rs4385909 | 11 | 124338543 | A | 3216 | 1.24 | 1.12-1.37 | 2.83e-05 | 2.98e-04 |
| 30 | rs4365066 | 11 | 124338595 | T | 3212 | 1.24 | 1.12-1.38 | 2.40e-05 | 2.62e-04 |
| 30 | rs4595557 | 11 | 124338664 | C | 3208 | 1.24 | 1.12-1.38 | 2.53e-05 | 3.03e-04 |
| 30 | rs4245055 | 11 | 124339489 | C | 3212 | 1.24 | 1.12-1.38 | 2.40e-05 | 2.62e-04 |
| 30 | rs11219669 | 11 | 124341481 | A | 3207 | 1.24 | 1.12-1.38 | 2.40e-05 | 2.62e-04 |
| 30 | rs10893231 | 11 | 124341722 | G | 3217 | 1.24 | 1.12-1.38 | 2.68e-05 | 2.07e-04 |
| 30 | rs2298509 | 11 | 124341985 | G | 3206 | 1.25 | 1.13-1.38 | 1.66e-05 | 1.81e-04 |
| 30 | rs10736541 | 11 | 124343714 | G | 3218 | 1.24 | 1.12-1.37 | 3.01e-05 | 3.39e-04 |
| 30 | rs1074873 | 11 | 124343736 | T | 3210 | 1.25 | 1.13-1.38 | 1.88e-05 | 2.61e-04 |
| 30 | rs10736542 | 11 | 124344365 | C | 3220 | 1.24 | 1.12-1.38 | 2.39e-05 | 2.17e-04 |
| 30 | rs10736543 | 11 | 124344789 | G | 3221 | 1.24 | 1.12-1.38 | 2.35e-05 | 2.13e-04 |
| 30 | rs10732863 | 11 | 124344940 | A | 3221 | 1.24 | 1.12-1.38 | 2.35e-05 | 2.13e-04 |
| 30 | rs10736544 | 11 | 124345248 | T | 3221 | 1.24 | 1.12-1.38 | 2.33e-05 | 2.13e-04 |
| 30 | rs10431090 | 11 | 124345602 | G | 3221 | 1.24 | 1.12-1.38 | 2.33e-05 | 2.13e-04 |
| 30 | rs10431091 | 11 | 124345791 | A | 3222 | 1.24 | 1.12-1.37 | 2.74e-05 | 2.42e-04 |
| 30 | rs10750278 | 11 | 124346851 | A | 3220 | 1.24 | 1.12-1.38 | 2.39e-05 | 2.16e-04 |
| 30 | rs7120864 | 11 | 124348499 | G | 3220 | 1.24 | 1.12-1.38 | 2.39e-05 | 2.17e-04 |
| 30 | rs10750279 | 11 | 124348959 | C | 3220 | 1.24 | 1.12-1.38 | 2.39e-05 | 2.17e-04 |
| 30 | rs10750280 | 11 | 124349120 | G | 3220 | 1.24 | 1.12-1.38 | 2.39e-05 | 2.17e-04 |
| 30 | rs4356237 | 11 | 124349711 | A | 3220 | 1.24 | 1.12-1.38 | 2.39e-05 | 2.17e-04 |
| 30 | rs9971477 | 11 | 124350167 | C | 3220 | 1.24 | 1.12-1.38 | 2.39e-05 | 2.17e-04 |
| 30 | rs9971552 | 11 | 124350467 | T | 3220 | 1.24 | 1.12-1.38 | 2.39e-05 | 2.17e-04 |
| 30 | rs9971496 | 11 | 124350579 | C | 3220 | 1.24 | 1.12-1.38 | 2.39e-05 | 2.17e-04 |
| 30 | rs11361196 | 11 | 124351076 | G | 3220 | 1.24 | 1.12-1.38 | 2.39e-05 | 2.42e-04 |
| 30 | rs4492834 | 11 | 124351409 | T | 3220 | 1.24 | 1.12-1.38 | 2.39e-05 | 2.41e-04 |
| 30 | rs10689492 | 11 | 124362896 | ACT | 3140 | 1.24 | 1.12-1.37 | 4.66e-05 | 5.90e-04 |
| 30 | rs4282991 | 11 | 124386346 | C | 3196 | 1.23 | 1.12-1.37 | 4.51e-05 | 2.67e-04 |
| 30 | rs10893236 | 11 | 124386739 | C | 3192 | 1.24 | 1.12-1.38 | 2.47e-05 | 2.79e-04 |
| **30** | **rs10893237** | **11** | **124386765** | **G** | **3173** | **1.26** | **1.14-1.39** | **1.15e-05** | 1.65e-04 |
| **31** | **rs116852744** | **11** | **125506599** | **C** | **3199** | **0.5** | **0.36-0.70** | **4.60e-05** | 6.79e-04 |
| **32** | **rs35681508** | **11** | **125765528** | **A** | **3219** | **0.49** | **0.36-0.68** | **1.86e-05** | 9.78e-04 |
| **33** | **rs12792252** | **11** | **130368580** | **A** | **3132** | **1.88** | **1.40-2.54** | **3.56e-05** | 4.69e-05 |
| **34** | **rs10745786** | **12** | **97592122** | **T** | **3204** | **1.27** | **1.13-1.42** | **3.45e-05** | 1.13e-04 |
| 35 | rs67604103 | 13 | 30856315 | A | 3161 | 1.24 | 1.12-1.38 | 4.65e-05 | 3.71e-05 |
| 35 | rs73163487 | 13 | 30857400 | C | 3161 | 1.24 | 1.12-1.38 | 4.71e-05 | 3.80e-05 |
| 35 | rs8000334 | 13 | 30857565 | T | 3161 | 1.24 | 1.12-1.38 | 4.71e-05 | 3.80e-05 |
| **35** | **rs10612266** | **13** | **30859460** | **T** | **3152** | **1.25** | **1.12-1.38** | **4.63e-05** | 3.72e-05 |
| 36 | rs7333521 | 13 | 81593016 | T | 3151 | 1.63 | 1.29-2.06 | 4.74e-05 | 3.05e-04 |
| **36** | **rs17073714** | **13** | **81609005** | **C** | **3178** | **1.66** | **1.31-2.10** | **3.25e-05** | 9.55e-04 |
| 37 | rs138667029 | 14 | 28075972 | C | 3103 | 1.7 | 1.32-2.18 | 3.49e-05 | 1.11e-04 |
| **37** | **rs61972524** | **14** | **28081832** | **G** | **3092** | **1.71** | **1.33-2.20** | **2.49e-05** | 6.39e-05 |
| 37 | rs61972525 | 14 | 28083229 | G | 3092 | 1.71 | 1.33-2.20 | 2.49e-05 | 6.39e-05 |
| **38** | **rs385803** | **14** | **59142509** | **A** | **3160** | **1.28** | **1.14-1.43** | **2.41e-05** | 1.01e-04 |
| **39** | **rs28514905** | **15** | **67056061** | **T** | **3138** | **1.44** | **1.22-1.70** | **2.08e-05** | 4.38e-05 |
| 39 | rs28461652 | 15 | 67056109 | T | 3138 | 1.44 | 1.22-1.70 | 2.08e-05 | 4.54e-05 |
| **40** | **rs4887377** | **15** | **88719499** | **C** | **3120** | **0.77** | **0.68-0.87** | **4.79e-05** | 2.44e-04 |
| **41** | **rs9937453** | **16** | **22155629** | **G** | **3200** | **1.37** | **1.19-1.59** | **1.61e-05** | 2.91e-06 |
| 42 | rs10852533 | 16 | 54846383 | G | 3159 | 1.36 | 1.18-1.57 | 2.49e-05 | 8.16e-05 |
| **42** | **rs6499721** | **16** | **54851878** | **G** | **3208** | **1.36** | **1.18-1.56** | **1.70e-05** | 9.57e-05 |
| 42 | rs7203670 | 16 | 54854466 | C | 3218 | 1.32 | 1.15-1.51 | 4.96e-05 | 2.56e-04 |
| 43 | rs80350164 | 17 | 43043640 | G | 3195 | 2.02 | 1.44-2.82 | 3.98e-05 | 6.41e-04 |
| **43** | **rs75990917** | **17** | **43068328** | **A** | **3165** | **1.81** | **1.37-2.40** | **3.55e-05** | 6.59e-04 |
| 43 | 17:43222659:G:C | 17 | 43222659 | C | 3153 | 2.07 | 1.51-2.84 | 6.41e-06 | 2.71e-04 |
| 43 | rs78420531 | 17 | 43249246 | T | 3113 | 1.61 | 1.30-1.98 | 8.74e-06 | 1.64e-04 |
| 43 | rs62066677 | 17 | 43254421 | A | 3099 | 1.46 | 1.22-1.74 | 4.10e-05 | 3.11e-04 |
| **44** | **rs17774211** | **18** | **71487700** | **A** | **3204** | **1.26** | **1.13-1.39** | **1.14e-05** | 9.60e-05 |
| **45** | **rs406475** | **20** | **38472799** | **G** | **3173** | **0.8** | **0.73-0.89** | **3.69e-05** | 2.65e-05 |
| 46 | rs35732478 | 20 | 60315652 | AC | 3210 | 1.91 | 1.40-2.59 | 3.46e-05 | 7.61e-04 |
| 46 | rs6061785 | 20 | 60316227 | T | 3206 | 1.89 | 1.39-2.55 | 3.85e-05 | 7.94e-04 |
| 46 | rs561587039 | 20 | 60317184 | CA | 3105 | 1.87 | 1.39-2.52 | 3.75e-05 | 5.37e-04 |
| 46 | rs13045675 | 20 | 60317789 | A | 3222 | 1.92 | 1.42-2.60 | 2.62e-05 | 5.97e-04 |
| 46 | rs34692315 | 20 | 60324231 | G | 3221 | 1.51 | 1.24-1.83 | 2.83e-05 | 2.80e-04 |
| 46 | rs60859095 | 20 | 60336865 | G | 3170 | 1.57 | 1.27-1.94 | 2.44e-05 | 1.82e-04 |
| **46** | **rs66805842** | **20** | **60337948** | **T** | **3168** | **1.58** | **1.28-1.95** | **2.04e-05** | 1.57e-04 |
| **47** | **rs5751846** | **22** | **24715049** | **A** | **3221** | **1.28** | **1.15-1.43** | **6.11e-06** | 8.46e-05 |

**Supplementary Table S5: Categorical analysis of PRS association to persistence to methotrexate across quintiles for the primary analysis cohort and its two subcohorts of seropositive and -negative RA patients.**

| **ASSOCIATION BETWEEN RA PRS AND PERSISTENCE TO MTX ACROSS PRS QUINTILES** | | | | | | |
| --- | --- | --- | --- | --- | --- | --- |
|  | **Persistence at one year** | | | **Persistence at three years** | | |
|  | **Overall RA** | **Seropositive RA** | **Seronegative RA** | **Overall RA** | **Seropositive RA** | **Seronegative RA** |
| **PRS ≤** $\boldsymbol{q}_{\boldsymbol{20}}$ | Ref | Ref | Ref | Ref | Ref | Ref |
| $\boldsymbol{q}_{\boldsymbol{20}}$ **< PRS ≤** $\boldsymbol{q}_{\boldsymbol{40}}$ | 1.04 (0.96-1.12) | 0.98 (0.89-1.07) | 1.15 (0.97-1.36) | 0.98 (0.87-1.11) | 0.99 (0.85-1.15) | 0.91 (0.71-1.17) |
| $\boldsymbol{q}_{\boldsymbol{40}}$ **< PRS ≤** $\boldsymbol{q}_{\boldsymbol{60}}$ | 1.01 (0.93-1.09) | 0.99 (0.90-1.09) | 1.03 (0.86-1.24) | 0.98 (0.87-1.11) | 1.00 (0.86-1.15) | 0.76 (0.59-1.00) |
| $\boldsymbol{q}_{\boldsymbol{60}}$ **< PRS ≤** $\boldsymbol{q}_{\boldsymbol{80}}$ | 0.94 (0.87-1.02) | 0.93 (0.84-1.03) | 0.86 (0.69-1.07) | 0.94 (0.83-1.06) | 0.95 (0.82-1.11) | 0.84 (0.64-1.11) |
| $\boldsymbol{q}_{\boldsymbol{80}}$ **< PRS** | 0.99 (0.91-1.07) | 0.93 (0.85-1.03) | 1.16 (0.98-1.37) | 0.93 (0.82-1.05) | 0.88 (0.75-1.03) | 1.01 (0.80-1.28) |
| **P-for-trend** | 0.1710 | 0.1603 | 0.1017 | 0.0966 | 0.7254 | 0.9459 |
| Parameters $q_{n}$ denotes the n%-quantiles of the standard normal distribution. | | | | | | |

**Supplementary Table S6: Cohort characteristics and results in the sensitivity analysis subcohort**. *Liability-scale heritability estimates and RRs quantifying the effect of the PRS on the persistence phenotypes, based on a sensitivity analysis sub-cohort of Swedish early RA patients, treated with MTX in DMARD-monotherapy as their first ordinated treatment, excluding patients included in SRQb more than 90 days after their initial MTX prescription.*

|  | | **Persistence at one year** | | **Persistence at three years** | |
| --- | --- | --- | --- | --- | --- |
|  |  | *Persistent*  *N =1569* | *Non-persistent*  *N = 779* | *Persistent*  *N = 1109* | *Non-persistent*  *N = 1289* |
| **Demographics** |  |  |  |  |  |
|  | *Female (%)* | 1063 (68%) | 577 (74%) | 738 (67%) | 882 (73%) |
|  | *Seropositive (%)* | 1052 (67%) | 554 (71%) | 739 (67%) | 846 (70%) |
|  | *Age (SD)* | 56 (13) | 53 (13) | 57 (13) | 53 (13) |
|  | *Year at MTX start (IQR)* | 2007 (04-12) | 2008 (05-12) | 2007 (04-12) | 2008 (05-12) |
| **Educational level** |  |  |  |  |  |
|  | *< 9 year (%)* | 384 (24%) | 168 (22%) | 282 (25%) | 260 (22%) |
|  | *9 – 12 years (%)* | 725 (46%) | 368 (47%) | 506 (46%) | 574 (48%) |
|  | *≥ 12 years (%)* | 458 (29%) | 242 (31%) | 319 (29%) | 373 (31%) |
| **Rheumatic disease comorbidity index** |  |  |  |  |  |
|  | *0 (%)* | 1217 (78%) | 630 (81%) | 861 (78%) | 967 (80%) |
|  | *1 (%)* | 128 (8%) | 60 (8%) | 83 (7%) | 101 (8%) |
|  | *≥ 2 (%)* | 120 (8%) | 53 (7%) | 86 (8%) | 79 (7%) |
| **Baseline disease activity components** |  |  |  |  |  |
|  | *SJC28 (SD)* | 9 (6) | 9 (5) | 9 (6) | 9 (5) |
|  | *TJC28 (SD)* | 8 (6) | 9 (6) | 7 (6) | 9 (6) |
|  | *CRP (SD)* | 21.47 (28.41) | 28.51 (35.43) | 21.38 (26.29) | 25.91 (34.69) |
|  | *ESR (SD)* | 30.27 (21.84) | 35.49 (24.4) | 29.99 (21.2) | 33.42 (24.02) |
|  | *Patient Global Health (SD)* | 46.04 (25.15) | 53.12 (25.31) | 44.73 (25.31) | 51.43 (25.05) |
|  | *DAS28 (SD)* | 5.01 (1.27) | 5.42 (1.24) | 4.96 (1.28) | 5.3 (1.26) |
| **MTX initiation characteristics** |  |  |  |  |  |
|  | *Starting dose 30mg (%)* | 273 (17%) | 139 (18%) | 200 (18%) | 203 (17%) |
|  | *Starting dose 100mg (%)* | 743 (47%) | 329 (42%) | 513 (46%) | 546 (45%) |
|  | *Other starting dose (%)* | 41 (3%) | 20 (3%) | 30 (3%) | 30 (2%) |
|  |  |  |  |  | |
|  | *Oral MTX (%)* | 1060 (68%) | 476 (61%) | 745 (67%) | 769 (64%) |
|  | *Folic acid supplementation (%)* | 1050 (67%) | 480 (62%) | 737 (66%) | 771 (64%) |
|  | *Prednisolone supplementation (%)* | 1274 (81%) | 676 (87%) | 883 (80%) | 1046 (87%) |
| **Liability-scale SNP-heritability** |  |  |  |  |  |
|  | *Crude h^2^ (SD)* | 0.30 (0.22) | | 0.32 (0.21) | |
|  | *Adjusted^†^ h^2^ (SD)* | 0.14 (0.25) | | 0.18 (0.23) | |
| **Polygenic risk score RR** |  |  |  |  |  |
|  | *Crude RR (95% CI)* | 0.98 (0.95 – 1.01) | | 0.96 (0.92 – 1.00) | |
|  | *Adjusted^†^ RR (95% CI)* | 0.98 (0.96-1.01) | | 0.97 (0.93-1.01) | |
| ^†^: Adjusted for age, sex and genetic ancestry per principal components.  CRP - C-reactive protein; DAS28 - disease activity score based on 28 joint counts; ESR - erythrocyte sedimentation rate; DMARD - disease-modifying anti-rheumatic drug; SJC28 - swollen joint count in 28 joints; TJC28 - tender joint count in 28 joints. | | | | | |

**Supplementary Table S7: Cohort characteristics and results in the supplementary analysis cohort.** *Cohort demographics and RRs quantifying the effect of the PRS on the persistence phenotypes, based on a cohort of Swedish early RA patients treated with MTX in DMARD-monotherapy included into after 2017.*

|  | | **Persistence at one year** | | **Persistence at three years** | |
| --- | --- | --- | --- | --- | --- |
|  |  | *Persistent*  *N = 447* | *Non-persistent*  *N = 187* | *Persistent*  *N = 273* | *Non-persistent*  *N = 240* |
| **Demographics** |  |  |  |  |  |
|  | *Female (%)* | 308 (69%) | 130 (70%) | 183 (67%) | 175 (73%) |
|  | *Seropositive (%)* | 123 (70%) | 55 (74%) | 87 (67%) | 88 (75%) |
|  | *Age (SD)* | 61 (15) | 56 (16) | 61 (14) | 56 (16) |
|  | *Year at MTX start (IQR)* | 2016 (14-17) | 2016 (14-17) | 2015 (13-17) | 2016 (13-17) |
| **Educational level** |  |  |  |  |  |
|  | *< 9 year (%)* | 86 (19%) | 44 (24%) | 57 (21%) | 50 (21%) |
|  | *9 – 12 years (%)* | 208 (47%) | 83 (44%) | 125 (46%) | 112 (47%) |
|  | *≥ 12 years (%)* | 149 (33%) | 57 (30%) | 89 (33%) | 73 (30%) |
| **Rheumatic disease comorbidity index** |  |  |  |  |  |
|  | *0 (%)* | 343 (77%) | 150 (80%) | 207 (76%) | 197 (82%) |
|  | *1 (%)* | 42 (9%) | 24 (13%) | 25 (9%) | 24 (10%) |
|  | *≥ 2 (%)* | 56 (13%) | 12 (6%) | 35 (13%) | 18 (8%) |
| **Baseline disease activity components** |  |  |  |  |  |
|  | *SJC28 (SD)* | 6 (5) | 7 (5) | 6 (5) | 6 (5) |
|  | *TJC28 (SD)* | 63 | 32 | 41 | 43 |
|  | *CRP (SD)* | 16.99 (21.12) | 23.94 (24.36) | 16.67 (21.08) | 21.4 (24.31) |
|  | *ESR (SD)* | 31.24 (24.43) | 31.91 (25.9) | 29.89 (23.26) | 31.55 (27.37) |
|  | *Patient Global Health (SD)* | 45.76 (25.31) | 51.61 (28.43) | 45.42 (25.15) | 51.01 (25.93) |
|  | *DAS28 (SD)* | 4.69 (1.39) | 4.94 (1.31) | 4.61 (1.46) | 4.8 (1.26) |
| **MTX initiation characteristics** |  |  |  |  |  |
|  | *Starting dose 30mg (%)* | 65 (15%) | 25 (13%) | 39 (14%) | 37 (15%) |
|  | *Starting dose 100mg (%)* | 274 (61%) | 120 (64%) | 158 (58%) | 145 (60%) |
|  | *Other starting dose (%)* | 54 (12%) | 22 (12%) | 34 (12%) | 32 (13%) |
|  |  |  |  |  |  |
|  | *Oral MTX (%)* | 391 (87%) | 164 (88%) | 230 (84%) | 212 (88%) |
|  | *Folic acid supplementation (%)* | 380 (85%) | 165 (88%) | 223 (82%) | 208 (87%) |
|  | *Prednisolone supplementation (%)* | 394 (88%) | 177 (95%) | 234 (86%) | 227 (95%) |
| **Polygenic risk score RR** |  |  |  |  |  |
|  | *Crude RR (95% CI)* | 1.01 (0.96 – 1.06) | | 1.02 (0.94 – 1.10) | |
|  | *Adjusted^†^ RR (95% CI)* | 1.01 (0.96 – 1.06) | | 1.02 (0.94 – 1.11) | |
| ^†^: Adjusted for age, sex and genetic ancestry per principal components.  CRP - C-reactive protein; DAS28 - disease activity score based on 28 joint counts; ESR - erythrocyte sedimentation rate; DMARD - disease-modifying anti-rheumatic drug; SJC28 - swollen joint count in 28 joints; TJC28 - tender joint count in 28 joints. | | | | | |

**REFERENCES**

1. Padyukov, L., et al., *A gene-environment interaction between smoking and shared epitope genes in HLA-DR provides a high risk of seropositive rheumatoid arthritis.* Arthritis Rheum, 2004. **50**(10): p. 3085-92.

2. SRQ. *SRQ Biobank*. 2022 [cited 2022 7/4]; Available from: <https://srq.nu/biobank-vardgivare/>.

3. Genomes Project, C., et al., *A global reference for human genetic variation.* Nature, 2015. **526**(7571): p. 68-74.

4. Howie, B.N., P. Donnelly, and J. Marchini, *A flexible and accurate genotype imputation method for the next generation of genome-wide association studies.* PLoS Genet, 2009. **5**(6): p. e1000529.

5. Patterson, N., A.L. Price, and D. Reich, *Population structure and eigenanalysis.* PLoS Genet, 2006. **2**(12): p. e190.

6. Chang, C.C., et al., *Second-generation PLINK: rising to the challenge of larger and richer datasets.* Gigascience, 2015. **4**: p. 7.

7. Purcell, S., et al., *PLINK: a tool set for whole-genome association and population-based linkage analyses.* Am J Hum Genet, 2007. **81**(3): p. 559-75.

8. Okada, Y., et al., *Genetics of rheumatoid arthritis contributes to biology and drug discovery.* Nature, 2014. **506**(7488): p. 376-81.

9. Prive, F., J. Arbel, and B.J. Vilhjalmsson, *LDpred2: better, faster, stronger.* Bioinformatics, 2020.
